# Monitoring single-cell gene regulation under dynamically controllable conditions with integrated microfluidics and software

**DOI:** 10.1038/s41467-017-02505-0

**Published:** 2018-01-15

**Authors:** Matthias Kaiser, Florian Jug, Thomas Julou, Siddharth Deshpande, Thomas Pfohl, Olin K. Silander, Gene Myers, Erik van Nimwegen

**Affiliations:** 1 Biozentrum, University of Basel and Swiss Institute of Bioinformatics, Klingelbergstrasse 50/70, 4056 Basel, Switzerland; 2 Max Planck Institute of Molecular Cell Biology and Genetics, Pfotenhauerstraße 108, 01307 Dresden, Germany; 3 Department of Chemistry, University of Basel, Spitalstrasse 51, 4056 Basel, Switzerland; 4 Present Address: Department of Bionanoscience, TU Delft, Van der Maasweg 9, 2629 HZ Delft, The Netherlands; 5 Present Address: Institute of Natural and Mathematical Sciences, Massey University Auckland, Private Bag 102904, North Shore, 0745 New Zealand

## Abstract

Much is still not understood about how gene regulatory interactions control cell fate decisions in single cells, in part due to the difficulty of directly observing gene regulatory processes in vivo. We introduce here a novel integrated setup consisting of a microfluidic chip and accompanying analysis software that enable long-term quantitative tracking of growth and gene expression in single cells. The dual-input Mother Machine (DIMM) chip enables controlled and continuous variation of external conditions, allowing direct observation of gene regulatory responses to changing conditions in single cells. The Mother Machine Analyzer (MoMA) software achieves unprecedented accuracy in segmenting and tracking cells, and streamlines high-throughput curation with a novel leveraged editing procedure. We demonstrate the power of the method by uncovering several novel features of an iconic gene regulatory program: the induction of *Escherichia coli*’s *lac* operon in response to a switch from glucose to lactose.

## Abstract (web summary)

How gene regulatory pathways control cell fate decisions in single cells is not fully understood. Here the authors present an integrated dual-input microfluidic chip and a linked analysis software, enabling tracking of gene regulatory responses of single bacterial cells to changing conditions.

## Introduction

Gene regulation is one of the key processes that underlie the complex behavior of biological systems, allowing cells to adapt to varying environments, and allowing multi-cellular organisms to express a large number of phenotypically distinct cell types from a single genotype. In spite of more than half a century of intense study since the discovery of the basic mechanism of gene regulation^1^, much remains to be understood about the ways in which gene regulatory interactions control cell fate decisions. Because of a number of challenges, it is still difficult to directly observe and measure gene regulation in vivo. First, gene regulation is inherently stochastic, and genetically identical cells in homogeneous environments often exhibit heterogeneous behaviors^2,3^. This implies that bulk expression measurements are often misleading, thus necessitating methods for studying gene regulation in single cells. Second, while methods such as flow cytometry, smFISH, and single-cell RNA-seq provide snapshots of gene expression distributions across single cells (see e.g. refs. ^3–5^), understanding the processes that shape these distributions often requires that single-cell gene expression be followed in time (e.g. refs. ^6,7^). The most common approach in such studies is to grow cells on a surface while tracking gene expression and growth using quantitative fluorescence time-lapse microscopy (QFTM).

Three key issues currently limit the power of such studies. First, to capture crucial regulatory events, long-term observations stretching over many cell cycles are often required. Second, measuring gene regulatory responses requires the ability to accurately control and vary environmental conditions. And third, to accurately characterize the statistics of single-cell responses, powerful image-analysis tools are needed to automatically extract large numbers of quantitative phenotypes from the time-lapse measurements. Considering bacteria, while it is possible to expose cells growing on surfaces to changing conditions^8–10^, gathering long time courses is not possible because the microcolonies grow out of the field of view or start to form multiple layers.

Recently developed microfluidic devices solve this problem by growing cells in micro-fabricated geometries that confine their location and movement^11–13^. An especially attractive design is the so-called Mother Machine^11^, in which cells grow single-file within narrow growth-channels that are perpendicularly connected to a main flow-channel that supplies nutrients and washes away cells extruding from the growth channels. However, all current designs expect a single media to be used as input, necessitating manual switching of the input to alter conditions, e.g. refs. ^14,15^, which precludes the accurate temporal control of the growth environment that is desired to study gene regulation in vivo.

In addition, beyond specific technical problems, many researchers are likely discouraged from studying gene regulation using a combination of microfluidics and time-lapse microscopy, because of the high costs associated with establishing the necessary methods. One not only needs to obtain designs for microfluidic devices, learn how to manufacture these, and work out experimental protocols for performing time-lapse experiments, one also needs sophisticated image-analysis and post-processing methods to obtain accurate quantitative information from the data. While there are a number of software tools for analyzing QFTM data of micro-colonies on agar^16–18^, they perform poorly on data from microfluidic devices such as the Mother Machine, because cells undergo larger movements between consecutive frames. In addition, phase contrast images in microfluidic devices often suffer from non-uniformity due to varying background and opacity. For this reason, most require a dedicated fluorescent reporter to assist segmentation. Although a number of labs are analyzing data from microfluidic devices using various inhouse image-analysis solutions^11,14,19–21^, there is currently no publically available tool that allows automated analysis of such data with the throughput and accuracy required for quantifying growth and gene expression in large data sets.

To address these problems, we here present an integrated experimental and computational setup for studying gene regulation in single cells using microfluidics in combination with time-lapse microscopy. Our approach consists of the combination of, first, a new microfluidic device, called the dual-input Mother Machine (DIMM), that allows arbitrary time-varying mixtures of two input media, such that cells can be exposed to a precisely controlled set of varying external conditions. Second, to enable high-throughput and high accuracy analysis of phenotypic measurements from the DIMM, we accompany it with a software suite, called MoMA (Mother Machine Analyzer). The Mother Machine Analyzer takes specific advantage of the geometry of the device to accurately segment and track cells using only phase-contrast images, and further provides a curation user interface with leveraged-editing, meaning that a set of related errors are often fixed with a single click. The combination of MoMA’s accuracy and curation efficiency allows analyses of data sets involving millions of single-cell observations. Third, we provide several methods for precise quantification and characterization of the accuracy of growth and gene expression measurements. By making the entire framework including the microfluidic device’s design, protocols for manufacture and time-lapse experiments, the open source MoMA software, and post-processing methods, all jointly available, we aim to dramatically lower the entrance costs for researchers to adopt this methodology. To demonstrate the power of the method, we apply it to the iconic *lac* operon regulatory system that underlies the discovery of gene regulation, and uncovers several novel unexpected features of its stochastic induction dynamics.

## Results

### The dual-input Mother Machine

**Fig. 1:**
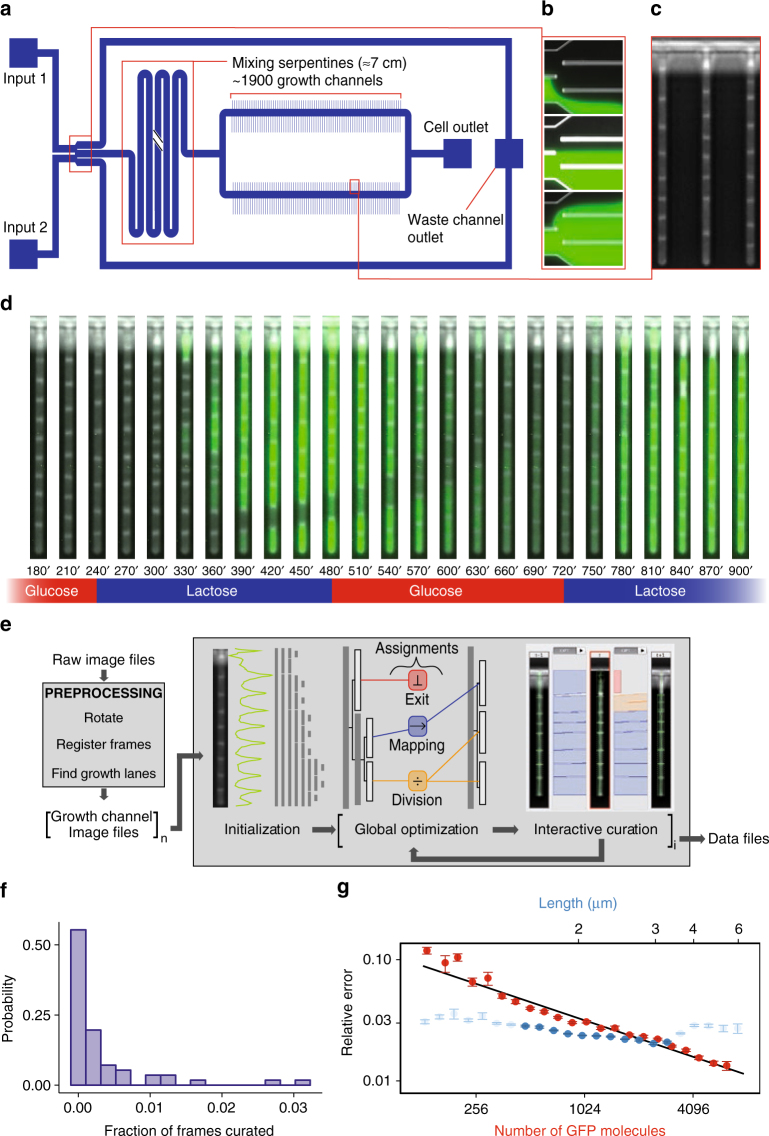
The dual-input Mother Machine. **a** Overview of the dual-input Mother Machine (DIMM) design. **b** Dial-a-wave junction in three different switching states, top: 100% from input 1 (unlabeled) and 0% from input 2 (green), middle: 50% from both inputs, bottom: 0% from input 1 and 100% from input 2. **c** Phase contrast image of growing *Escherichia coli* cells in three growth-channels of the DIMM. **d** A time series of a single growth-channel containing *E. coli* cells expressing LacZ-GFP from the *lac* promoter while being exposed to media which alternate between containing glucose and lactose as a carbon source. **e** Overview of the automated and curation phases of the MoMA analysis pipeline. **f** Histogram of the fraction of curated frames per single growth-channel time course. **g** Estimated measurement errors on cell size (blue) and number of GFP molecules (red). Dark blue points indicate the typical range of cell sizes. Error bars show standard errors. The black line shows the fitted function $1.01{\mathrm{/}}\sqrt {\left( x \right)}$

The design of our DIMM device closely follows that of the original Mother Machine^11^, consisting of a main channel and small dead-end growth channels that open into the main channel (Fig. 1a, c). Nutrients diffuse from the main channel into the growth-channels in which cells are trapped (Fig. 1c), and as the cells in the growth-channels grow and divide, cells closest to the channel’s exit are pushed out and are transported away by the flow in the main channel. In contrast to previous designs, our device has dual-input ports and mixing serpentines which, in combination with programmable pumps, allow for arbitrary time-dependent mixing of two input media. The two inputs meet in a dial-a-wave junction^22^ consisting of two inlets and three outlets (Fig. 1b). While the middle outlet feeds into the main channel of the device, the outer outlets function as waste channels and allow the flow in the middle outlet to vary from carrying only one of the two inputs (black in Fig. 1b), to carrying only the other input (green in Fig. 1b), without getting backflow into the inactive inlet. Note that arbitrary mixtures of the two input media are possible (see Methods, Performance of the environmental control) so that, for example, dynamically changing concentrations of particular nutrients or stressors can be realized. Details on the loading of the DIMM are provided in the Methods (Priming and loading of the microfluidic devices).

To demonstrate the power of our approach, we applied it to the archetypical example of a gene regulatory system: the induction of *Escherichia coli*’s *lac* operon when switching between glucose and lactose as a carbon source. We used a modified *E. coli* MG1655 strain that carries a translational *lacZ-gfp* (green fluorescent protein) fusion at its native locus^7^. Time lapse movies of 22−24 h were obtained in duplicate for three different setups (1. a constant supply of M9 minimal media+0.2% glucose, 2. a constant supply of M9+0.2% lactose, and 3. switching between these two media every 4 h), taking a frame every 3 min (see Supplementary Movie 1 (https://www.youtube.com/watch?v=2Tznm868fmc (2015))). Together with additional control conditions (strain without GFP, and switching media where lactose is supplemented with 500 μm IPTG (isopropyl β-D-1-thiogalactopyranoside), we thus analyzed eight different time-lapse experiments all together, amounting to data from 180 growth-channels, more than 10,000 full cell cycles, and more than 500,000 single-cell observations (Supplementary Table 1).

### Image analysis and data processing

The analysis of the image sequences acquired by a DIMM is performed in three phases by the MoMA software suite (see Methods, The Mother Machine Analyzer, and following sections). Although MoMA, by default, uses phase contrast images to segment and track the cells, leaving all fluorescent channels for measurement of gene expression and allowing tracking on non-fluorescent (e.g. wild-type) cells, the user can opt to let MoMA use fluorescence images for tracking. The first automated phase begins by registering the frames of a movie to sub-pixel accuracy to correct for jitter and stage drift. Then the growth-channels in each time frame are cropped out and reorganized into a time-series for each channel. Each growth-channel movie is then segmented and tracked. Unlike most image analysis tools that first segment each of the images and then link these segmentations into a tracking, MoMA uses an algorithm that first over-predicts a hierarchy of feasible cell objects (segmentations) for each time point and then simultaneously selects what it thinks are the true cells and the tracking links between them^23^. This is accomplished by formulating prior information as a collection of integer linear constraints that guarantee only valid cell trackings satisfy the constraints, and finding among this space of valid trackings, the one of minimum cost. Since cost reflects the likelihood of the solution considering both the observations and prior constraints, this is equivalent to finding the maximum a posteriori solution in Bayesian statistics. We use Gurobi, a potent off-the-shelf integer linear program solver to do so (see Methods).

In the second curation phase, an interactive graphical user interface is opened that allows users to browse the results, identify errors, and correct them. In contrast to existing methods, where data curation is performed by directly editing the segmentations or linking graphs, MoMA offers various possibilities to browse through alternate segmentation hypotheses and tracking paths. Once a user makes an adjustment, e.g. by selecting an alternative segment or link, MoMA formulates the user’s choice as an additional constraint and restarts the optimization in order to find the new optimum solution incorporating this constraint. In this way corrections automatically percolate to nearby time points, typically fixing multiple mistakes at once. For the individual growth-channels of the 22–24 h time courses analyzed here, an average 0.3% of frames required a curation directive, and roughly half of the growth-channels required no curation at all (Fig. 1f). In our hands, it typically takes 1–2 min to curate 100 frames (see Methods, Curations statistics).

In the final quantification phase, we developed methods to quantitate the sizes of cells and the amount of fluorescent reporter, as well as to quantify the size of the errors on these measurements. When growing in a constant environment, cell sizes across the cell cycle closely follow an exponential growth curve in both conditions (median squared-correlation *R*^2^ ≈ 0.99, see Methods, Cell size and growth rate estimation) and this allows us to estimate an upper bound on the errors of individual size measurements, which we find to be approximately 3% (Fig. 1g, and see Methods, Cell size and growth rate estimation). Growth rates of individual cell cycles can be estimated within an error of 1–3% and we find average growth rates of 0.75 (glucose) and 0.69 (lactose) doublings per hour, which vary by 17% across cells (see Methods, Cell size and growth rate estimation). Growth rates during the lactose and glucose phases of the switching conditions have virtually the same distribution as in the corresponding constant conditions (see Methods, Cell size and growth rate estimation).

We observed that cell fluorescence spreads significantly beyond the cell, approximately as a Cauchy distribution as a function of distance from the cell, and we use a Bayesian mixture model to accurately estimate the fluorescence of a given cell (see Methods, Cell fluorescence estimation). This procedure removes auto-fluorescence due to the PDMS (polydimethylsiloxane) but not the auto-fluorescence of the cell and media. Using measurements on wild-type cells, we observed that auto-fluorescence is proportional to cell size and used this to subtract the contribution of auto-fluorescence to GFP fluorescence measurements (see Methods, Cell auto-fluorescence estimation). Finally, to estimate the conversion factor between fluorescence level and the number of GFP molecules we adapted the method of Rosenfeld et al.^24^ which is based on the assumption that fluctuations in the fluorescence levels of two daughter cells immediately after division derive from random binomial partitioning of the mother’s GFP molecules to the two daughters. We substantially improve on this method by (a) taking advantage of the DIMM design to use data only from the glucose phases in which no GFP synthesis occurs, (b) incorporating the slow fluorescence decay due to bleaching and protein decay (see Methods, Estimating GFP's bleaching and degradation), and (c) taking into account that fluctuations in the sizes of the daughters contribute significantly to the fluorescence fluctuations. We integrated all this into a Bayesian procedure and determined the posterior distribution of the conversion factor between fluorescence and number of LacZ-GFP tetramers (see Methods, Estimating the conversion factor between fluorescence and number of GFP molecules). Using this we find that, when growing in lactose, cells contain 3000–6000 GFP molecules at birth and 6000–12,000 GFP molecules just before division. Finally, we estimated the measurement errors of individual GFP measurements by quantifying the deviations of measured GFP levels from a simple exponential decay during the glucose phases of the switching experiment. In contrast to the relative error on size estimates, which are approximately independent of absolute size, we find that the error on GFP molecule number *G* scales as $1{\mathrm{/}}\sqrt G$ (Fig. 1g), which suggests that this measurement error is likely dominated by shot noise.

One problem encountered with sophisticated image analysis for cell tracking is that methods often poorly generalize to data from setups other than the specific one used in developing the methods. However, MoMA’s novel approach in which segmentation and tracking are treated as a joint optimization problem under a system of constraints ensures a high level of robustness to changes in the setup. To directly demonstrate MoMA’s general applicability, we reached out to MoMA’s emerging user community and obtained time-lapse data sets that were produced in other labs, using different microfluidic devices, different strains and species of bacteria, and different growth conditions (Supplementary Table 2). We confirmed that MoMA shows excellent performance on these data sets, both in terms of the needed curation interactions (Supplementary Fig. 1), and the quality of the resulting growth curves (Supplementary Fig. 2). We find that, depending on strains and conditions, growth rate fluctuations range between 10 and 20% of the average growth rate (Supplementary Fig. 3), and that the accuracy of estimated growth rates is determined to a large extent by the number of measurements per cell cycle (Supplementary Fig. 3).

### Single-cell dynamics of the *lac* operon

**Fig. 2:**
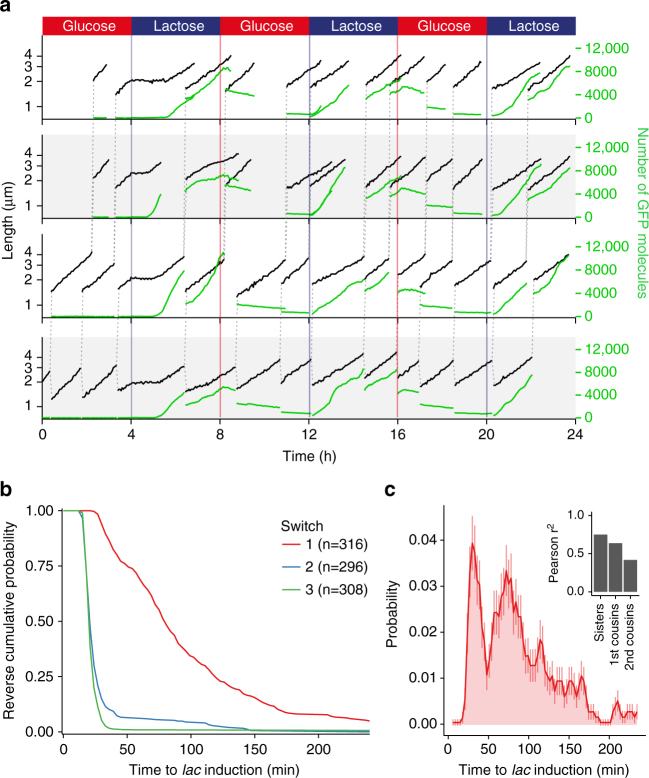
Tracking single-cell dynamics of *lac* operon induction. **a** Dynamics of growth and gene expression in lineages of single cells in an environment that switches between M9+0.2% glucose and M9+0.2% lactose every 4 h. Cell size (black, logarithmic scale) and expression of LacZ-GFP (green, linear scale) are shown as a function of time for a lineage of cells at the bottom of the growth-channel (bottom row) together with first-generation offspring and second-generation offspring cells (second row from the bottom, and top two rows, respectively). The dashed vertical lines show the lineage of cell divisions by connecting each mother cell to its two daughter cells. **b** Reverse cumulative distributions of lag times to LacZ-GFP induction in single cells at the first (red), second (blue), and third (green) switch from glucose to lactose. **c** Estimated probability distribution (mean and standard deviation) of single-cell lag times for the first switch in 3-min intervals. The inset shows the correlation in lag times for pairs of cells that had the same mother 1, 2, or 3 generations in the past

Figure 2a illustrates how our methodology allows accurate tracking of growth and gene expression across lineages of single cells as the environment is varied. As an example application, we focused our analysis on the single-cell dynamics of *lac* operon induction. Even before the discovery of gene regulation, it was already known that the induction of the *lac* operon is stochastic, with different single cells inducing at different times^25^. Further support for the stochasticity of this system has come from studies showing that, when exponentially growing populations are treated with artificial inducers such as IPTG or TMG (methyl-β-D-thiogalactoside), population snapshots often show a bimodal distribution of *lac* expression in single cells. The current consensus is that, in order for a cell to switch from a low expression to a high expression state, a sufficiently large stochastic burst of *lac* operon expression is needed^26–28^. A first attempt to measure the distribution of *lac* induction lag times in single cells was made by Boulineau et al.^10^, and a wide distribution of lag times was observed. However, the lack of a precise control of the growth media in that work not only precluded accurate time resolution of the lag times, but also caused the switch from glucose to lactose to be so gradual that only some cells experienced a transient reduction in growth rate, while others continued without any change in growth rate.

In contrast, we find that upon a controlled sudden switch from glucose to lactose, the effect on growth is not stochastic at all: all cells completely arrest growth within 3 min of the switch (Fig. 2a). Other aspects that are extremely reproducible are the fact that all cells exit growth arrest as soon as LacZ-GFP is at detectable levels (i.e. 100−200 molecules), and that LacZ-GFP production is halted almost immediately after switching back to glucose (Supplementary Fig. 4). Thus, while induction of the *lac* operon is highly stochastic, its shutdown and the coupling of growth to *lac* expression appears essentially deterministic.

Interestingly, while it might have been expected that, after exiting growth arrest, initial growth rates would be low when LacZ-GFP levels are still far below steady-state levels, we find that cells immediately grow at rates that are close to those observed in constant lactose, and reach steady-state growth rates within an hour of induction (Supplementary Fig. 5). We estimated instantaneous growth rate as a function of LacZ-GFP concentration and found only a substantial decrease when concentration is more than 10-fold below the steady-state levels of 2000–3000 molecules per μm of cell length (Supplementary Fig. 5). That is, cells can sustain high growth rates in lactose with *lac* operon expression that is fivefold or more below steady-state levels, in line with previous observations^10^. This raises the question as to why LacZ steady-state levels are so much higher than required for growth. One intriguing possibility is that such high expression levels allow for a memory of growth in lactose that lasts over several generations, something that has been observed previously at the population level^13^. Indeed, during the glucose phase the total fluorescence in each cell shows a slow exponential decay, mostly due to bleaching, and approximately halves at each cell division (Fig. 2a). By the time of the second switch to lactose, LacZ-GFP levels have diluted back to low levels, but the remaining *lac* expression is enough to ensure that all progeny of cells that induced in the first switch continue growth without an obvious decrease in growth rate, and quickly recommence LacZ-GFP production (Supplementary Fig. 6).

Our methodology allows, for the first time, the accurate measurement of the distribution of lag times for single cells to exit their growth arrest after the first switch from glucose to lactose. We not only observe a wide distribution of lag times, but find that this distribution is multi-modal: 27% of cells induce within 25–45 min, 68% induce within 50–240 min, and 5% of cells do not induce at all (Fig. 2b, c). Importantly, this observation is directly at odds with the current view in the literature that all lags are determined by the waiting time to a single stochastic event. Instead, the multi-modal distribution suggests that naive cells can exist in different states that determine their ability to respond to lactose.

We investigated whether lag times correlate with simple physiological quantities such as growth rate, cell cycle stage, or LacZ-GFP levels at the time of the switch, but found that none of these variables correlate with lag times (Supplementary Fig. 7). However, we find strong correlations of the lag times of cells that had the same mother, grand-mother, or even great-grandmother cell (Fig. 2c and Supplementary Fig. 8). It is especially striking that these genealogical correlations are larger for lag time than for any other physiological quantity that we measured, including quantities such as cell size and LacZ-GFP concentration, that are known to be directly inherited from the mother (Supplementary Fig. 9). In particular, only lag time shows significant correlations in cousins and second cousins. These results strongly suggest that lag time is controlled by an inheritable epigenetic factor that, in contrast to other physiological quantities such as LacZ-GFP expression, growth rate, and cell size, shows significant correlations over 2–3 generations.

Although a full investigation of the mechanistic interpretation of the multi-modal lag time distribution is beyond the scope of this work, we can propose an interpretation that we consider most plausible. We propose that the first and second modes of the lag distribution correspond to cells that, at the time of the switch, have either nonzero expression of both LacZ-GFP and LacY permease, or zero expression of either of these molecules. When both LacY and LacZ-GFP molecules are present at the switch, lactose can presumably immediately enter the cell, where it is metabolized into allolactose, causing *lac* operon derepression and LacZ-GFP production. In contrast, when no LacY/LacZ-GFP is present, lactose can either not enter the cell, or it cannot be metabolized, and cells first have to wait for a stochastic burst of *lac* operon expression, causing a longer lag time. If this interpretation is correct, then no long lags should be observed when an artificial inducer is added that can diffuse into the cell without LacY permease and binds directly to the LacI repressor. Indeed, when we add IPTG to the medium containing lactose we only observe short lags (Supplementary Fig. 10). Our interpretation also requires that, when growing in glucose, the majority of cells should contain either no LacY or no LacZ-GFP at all. This prediction is consistent with our LacZ-GFP measurements in glucose that predict the distribution of lacZ-GFP per cell significantly overlaps zero molecules (Supplementary Fig. 11). It is also broadly consistent with previous observations that, in similar growth conditions, roughly 50% of cells contain no LacY^27^, and 65% of the cells contain no LacZ^29^. Finally, we note that Choi et al.^27^ estimated that, when growing in the absence of lactose, small bursts in which around 40 LacY molecules are produced occur every 2–3 cell cycles, which is consistent with the waiting times of up to 240 min that we observe for cells of the second mode of the distribution.

## Discussion

We have here presented an integrated experimental and computational setup for quantifying gene expression dynamics at the single-cell level over long periods of time in dynamically changing environments that are precisely controlled. This methodology opens up a wide array of possibilities for studying gene regulatory mechanisms at the single-cell level. A single experiment with our setup was able to uncover several novel and unexpected features of one of the most intensely studied regulatory systems: *lac* operon expression under growth conditions that change between glucose and lactose as a carbon source. However, the technology enables many other types of investigations, e.g. it can be used to quantify how expression fluctuations affect growth rates at the single-cell level, to investigate how regulatory responses depend on the concentration and length of exposure to an inducing nutrient or stress, and how memories of regulatory responses are maintained across lineages of cells. More generally, its power extends beyond the scope of gene regulation studies. For example, it is becoming increasingly appreciated that single-cell heterogeneity plays an important role in persistence and evolution of resistance to antibiotics, and our methodology could be used to accurately quantify how single-cell growth and survival varies as a function of both the concentration and time of exposure to particular antibiotics, and as a function of the physiological states of the cells. In summary, we believe that the integrated experimental and computational methodology that we present here will be an important tool for studying gene regulatory mechanisms at the single-cell level. As detailed in the Data Availability section below, to facilitate access of other labs to our integrated methodology, we have collected all relevant information in a web repository, including files with the CAD designs of the device, information on fabrication of the device, detailed protocols for running the experiments, and links to the open source MoMA software. MoMA and its documentation, including tutorial videos are available online^30^ and, to make MoMA easily available to any user of ImageJ, we have also made MoMA available as a Fiji plugin.

## Methods

### Design and fabrication of the microfluidics devices

*Escherichia coli* cells take on different sizes depending on the media they are grown in, e.g. LB versus M9 minimal medium. Since the growth-channels aim to trap the cells growing in single file, the width of the channels needs to match the width of the cells as closely as possible. To account for this, our DIMM device contains channels with a range of widths, ranging from 0.8 to 1.6 μm, and lengths of 25 μm on one side of the device, and 55 μm on the other. For the results presented here, the growth-channel sections were ~0.9 μm (height) x ~0.8 μm (width), and 25 μm (length). These dimensions worked nicely with cells growing in M9+0.2% glucose or 0.2% lactose respectively. Experiments with other media and strains might require slightly different dimensions. In order to reduce the flow rates compared to the original mother machine device, the dimensions of the main channels were reduced to a diameter of 6 μm (height) by 50 μm (width) in the device presented here. The resulting flow rates are discussed in more detail in the section discussing loading and flow control. We note that reflections from the PDMS in the main channel can affect the phase contrast images near the top of the growth-channels, such that a segment of the growth-channels nearest to the exit needs to be discarded. To minimize this effect it is advisable to keep the main channel relatively shallow.

The device was designed using AutoCAD^®^ (AUTODESK^®^) and is freely available at Metafluidics, an open repository for fluidic systems^31^. We outsourced both the production of the photomask and the production of the masters to pour the PDMS devices from. A 5″ quartz mask with chrome layer was ordered from the Compugraphics Jena GmbH. Using this mask, Microresist (Berlin) produced the master using UV-lithography with SU-8 photoresists (for more details see ref. ^11^). To make the chips, we use the Sylgard Elastomer Kit 184 with a 1:10 curing agent to base ratio. Curing was performed at 65 °C overnight or longer. Harris Uni-Core 0.75 mm biopsy punches were used to create in- and outlets. Before bonding, both the PDMS mold and a cover slip were washed with isopropanol and dried with air. Surface activation was done in a plasma cleaner (PDC-32G, Harrick Plasma) operated at high intensity with vacuum at 1500 mTorr for 40 s. After bonding the devices were incubated at 65 °C for at least 1 h.

### Performance of the environmental control

The design presented here not only allows switching between different media but also allows for continuous control over the ratios of two different input media. Because flows in micro-channels are strictly laminar, only diffusive mixing occurs at these scales^22^. To keep the design simple we introduced 2D mixing serpentines to the device. These serpentines guarantee that the media coming together in the junction are flowing together long enough to allow for diffusive mixing before the mix reaches the cells. The required length of these mixing serpentines depends on the flow speed (fluid velocity), the width of the micro-channels, and the diffusion coefficient of the molecule of interest in the medium used^32^.

**Fig. 3:**
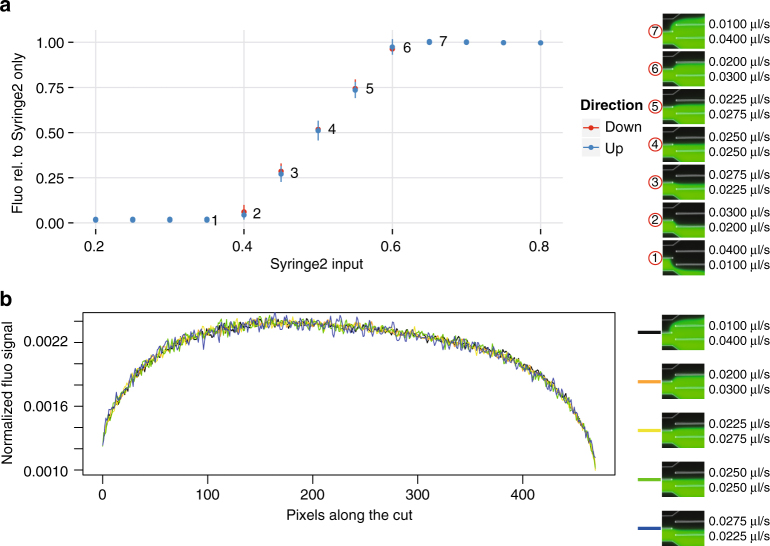
Mixing of fluorescein-labeled medium with non-labeled medium at different input flow rate ratios. **a** Total fluorescence was measured in a square region in the main channel downstream of the mixing serpentines as the input ratio was changed in a stepwise manner from 0% fluorescein input to 100% fluorescein input (up: blue) and back to 0% fluorescein input again (down: red). The fluorescence measured for the mixture relative to the fluorescence measured for pure fluorescein-labeled medium (Syringe 2 only) is plot against the input of Syringe 2 as a fraction of the total input from both Syringes 1 and 2. **b** Normalized fluorescence along a section through the main channel downstream of the mixing serpentines at different mixing ratios

To demonstrate mixing in the device we used M9 minimal medium labeled with fluorescein (1 g/ml) (Syringe 2) mixed with unlabeled M9 minimal medium (Syringe 1). We first obtained a reference fluorescence level for the medium containing fluorescein by measuring fluorescence every 15 s for 70 min, and taking the average of these 280 measurements. For 13 different relative flow rates of the two syringes, ranging from 20% of the total flow from Syringe 2 to 80% of the total flow from Syringe 2, we then measured fluorescence every 15 s for 10 min (40 min) and divided this by the reference fluorescence level to obtain a relative fluorescence. We then calculated the mean and standard deviation of 40 relative fluorescence levels for each relative flow rate. The results are shown in Fig. 3a, demonstrating how the system presented here can generate different mixing ratios and thus can be used to precisely control the growth environment. Figure 3b shows the normalized fluorescent intensity along a section through the main channel downstream of the mixing serpentines at different flow regimes. Because of small imperfections in the mold the intensity profile is not perfectly symmetrical even in the unmixed state (black line). However in the different mixed states, the shape of the profile stays the same indicating complete mixing is guaranteed in the flow regimes tested here.

### Strains and growth conditions

Strains were streaked from freezer stocks onto LB plates before experiments. Overnight precultures were grown from single colonies in M9 minimal medium supplemented with the same carbon source that the cells were to experience in the initial phase of the experiment (0.2% glucose or 0.2% lactose). The next day, cells were diluted 100-fold into fresh medium with the same carbon source. Cells were harvested after 4–6 h to be concentrated and loaded into the microfluidic device (typically, a culture at OD ≈ 0.2 was concentrated 100-fold). Growth occurred at 37 °C for both the precultures and the microscopy experiments. The growth conditions used during the microscopy experiments are described in Table 1.

**Table 1:** Experimental conditions used in this study

**Condition**	**Sequence of growth media**	**Strain**
No GFP	12 h: M9+0.2% glucose	MG1655
	12 h: M9+0.2% lactose	
Glucose	22 h: M9+0.2% glucose	ASC662
Lactose	22 h: M9+0.2% lactose	ASC662
Switch	4 h: M9+0.2% glucose	ASC662
	4 h: M9+0.2% lactose	
	4 h: M9+0.2% glucose	
	4 h: M9+0.2% lactose	
	4 h: M9+0.2% glucose	
	4 h: M9+0.2% lactose	
Switch IPTG	4 h: M9+0.2% glucose	ASC662
	4 h: M9+0.2% lactose+500 μm IPTG	
	4 h: M9+0.2% glucose	
	4 h: M9+0.2% lactose+500 μm IPTG	

Strain MG1655 is a reference K12 strain^46^, and ASC662 was derived from it by integrating a translational fusion *lacZ-gfp* in the native *lac* operon^7^. Note that for each condition, the first step of its sequence of growth media was preceded by 2 h in the same media (in order to reach growth steady-state under fluorescence illumination conditions) that were discarded from the data analysis

### Priming and loading of the microfluidic devices

**Fig. 4:**
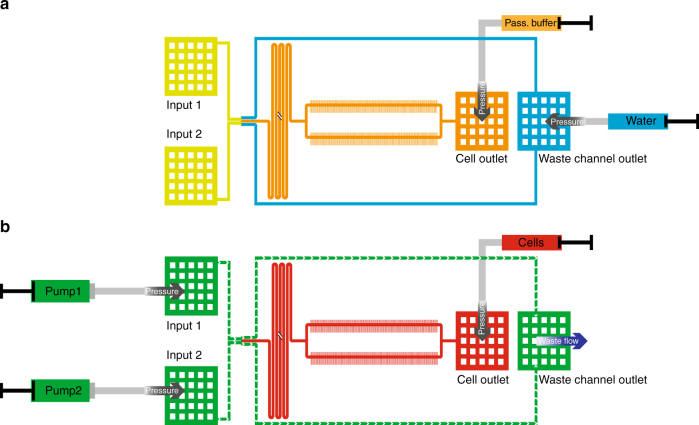
Priming and loading of the device. **a** Passivation buffer loading. To prevent blocking of the waste channels by passivation buffer, the waste channels are loaded with water through the waste channel outlet (blue) while loading of passivation buffer is done through the cell outlet (orange). Putting both outlets under pressure assures complete loading of the main channel with passivation buffer while the waste channels stay clear of passivation buffer. **b** A constant flow in both inlets (input 1 and input 2) prevents cells entering the inlets during the loading. The concentrated cell solution can be loaded through the cell outlet. First some pressure is applied to fill the whole main channel with cells. Afterwards the pressure is controlled to maintain zero flow in the main channel (red) while there is a constant flow through the inlets and in the waste channels (green) to remove cells that make it up to the dial-a-wave junction

The DIMM design presented here has two inlets and two outlets. This leads to some complications in the cell loading process compared to the original Mother Machine design. Here we describe the adjusted loading procedure we developed. As described in ref. ^11^, a mixture of salmon sperm DNA (10 mg/ml) and BSA (bovine serum albumin, 10 mg/ml) (at a ratio 1:3) is used to passivate the device before loading the cells. The salmon sperm DNA is denatured at 95 °C for 10 min and is mixed with the BSA after cooling down. This passivation buffer is also added to the growth medium in the experiment in a concentration of 1/100. In addition, one medium was always labeled with non-fluorescent microspheres (Polybead^®^ polystyrene 1 μm beads) to monitor medium flow at the dial-a-wave junction. As shown in Fig. 4a, the two dial-a-wave waste channels cannot be pressured separately because they both end in the same outlet. Therefore to prevent blockage of one of the waste channels by passivation buffer it is recommended to flow water into the waste channel outlet while the passivation buffer is loaded into the cell outlet. Once the main channel (with the growth-channels) is filled with passivation buffer and the inlets (input 1 and input 2) are full of liquid (mixture of water and passivation buffer), both the flow of water and of passivation buffer can be stopped. The device is now incubated for ca. 1 h at 37 °C before the loading of the cells is started.

After the passivation step, cell loading can begin. To get rid of the passivation buffer, the two inlets are connected to the pumps with the two different media that will be used in the experiment. At this point the tubing for the waste outlet can also be installed and connected to a waste container. Both pumps are now set to a flow rate of 1.5 μl/min. When all channels are clear, this flow regime will lead to a 50:50 ratio between the two inputs at the dial-a-wave junction. If the device leaks at this point or fails to establish a 50:50 ratio at the dial-a-wave junction (one medium is labeled with beads to check the flow under the microscope), most likely the resistance of some channel is altered by a blockage and the device cannot be used. If the device works properly, the dial-a-wave junction can be switched to the medium that will be used first. This step is necessary to ensure that the cells that are loaded afterwards only encounter the media condition in which they will begin growth. For a complete switch we use flow rates of 0.6 μl/min for the inactive inlet and 2.4 μl/min for the active one (Fig. 3a). After a few minutes (depending on the flow rate) the main channel and cell outlet should be free of the medium from the initial input and the cell loading process can begin. The cells are harvested in exponential phase and are concentrated by centrifugation (~100−200×). Once the device is fully switched to the desired input, one can load the cells using a 1 ml syringe into the tubing that will later serve as the waste tube. This tubing is inserted into the cell outlet and can be pressured by hand to flow the cells into the main channel (the loading process is observed under the microscope). It is important not to stop the flow at the inlets during the whole loading process. This allows cell loading without getting cells into the inlets where they might become stuck and grow. Once the cells reach the growth-channels we used a custom-made clamp to hold a precise level of pressure on the 1 ml syringe for cell loading. The pressure here has to be continuously adjusted to make sure the cells stop flowing in the main channel and can enter the growth-channels. As shown in Fig. 4b there is a constant flow through the inlets and the waste channels (green) while the main channel is pressured to achieve zero flow where the growth-channels are (red). If cells move up to the dial-a-wave junction they are removed through the waste channels and the inlets stay clear. Loading continues until a satisfactory number of channels contain cells (typically 20−60 min). When complete, the 1 ml syringe used for loading is removed, and the end of its tubing is put into the waste container together with the tubing from the waste channel outlet. After loading the cells are allowed to recover for at least 2 h before the experiment starts.

Growth media are delivered from air-tight glass syringes (Hamilton) that are connected to the device using PTFE tubing with an inner diameter of 0.56 mm and an outer diameter of 1.07 mm. The syringes are controlled by two low pressure pumps (Cetoni GmbH) so that the total flow during the experiment is 3 μl/min.

### Microscopy and data preprocessing

An inverted Nikon TI-E microscope equipped with a motorized xy-stage with linear encoders was used to perform the experiments. All experiments were performed in an incubator maintained at 37 °C. The sample was fixed on the stage using metal clamps and focus was maintained using the Perfect Focus System from Nikon. Images were recorded using a CFI Plan Apochromat Lambda DM ×100 objective (NA 1.45, WD 0.13 mm) and a CMOS camera (Hamamatsu Orca-Flash 4.0). The setup was controlled using Micro-Manager^33^ and timelapse movies were recorded with its Multi-Dimensional Acquisition engine (customized using runnables). Every 3 min one phase contrast image and one GFP fluorescence image were acquired, typically for six different positions. Phase contrast images were acquired using 100 ms exposure with the transmitted light source at full power (CoolLED pE-100). Images of GFP fluorescence were acquired using 2 s exposure, illuminating the sample with a Lumencor SpectraX (Cyan LED) set to 17% and dimmed using a ND4 filter in the light path; the excitation (475/35 nm) and emission filters (525/50 nm) were used with a dichroic beam-splitter at 495 nm. For the switching experiments images of the dial-a-wave junction were also acquired. Here the GFP channel was replaced with an additional phase contrast image with a short exposure time (10 ms) to visualize the beads in the flow.

The MoMA tracking software expects to be given image data sets in which a single growth-channel is present, with the growth-channel opening at the top, and with phase contrast being the first channel. With our microfluidic design, the camera field of view covers ca. 30 growth-channels so the images must be split into individual growth-channels and preprocessed in order to match MoMA’s requirements. The preprocessing consists of the following tasks:

1. Load the microscopy data set, one position at a time, in a format-independent manner using the Bio-Formats library (in order to open a specific position, one must use the Java API rather than functions available in ImageJ).
2. Register all frames to the first frame of the first channel in order to correct the sample drift over time, as well as the jitter introduced by acquiring multiple positions in parallel. To do this, we develop HyperStackReg, a custom extension of the StackReg ImageJ plugin that is able to handle hyperstacks, i.e. data sets with several channels.
3. Crop the image to keep only the area of the growth-channels and rotate the images (so that the growth-channel opening is at the top).
4. Save images as a tiff data set with one file per frame.
5. Straighten the image so that growth-channels are oriented vertically (using bicubic interpolation).
6. Identify the growth-channels in the first-phase contrast frame and save one data set per cropped growth-channel.

All steps are run in Fiji with the help of two utility plugin released together with MoMA: HyperStackReg and MMPreprocess. This preprocessing step is documented extensively on MoMA’s Wiki^30^, including how to run it from the command line. Note that in order to preprocess data sets from the command line, Fiji must be run using a virtual window environment (using Xvfb), since the headless mode is not compatible with some important ImageJ features.

### The Mother Machine Analyzer

Today’s predominant tracking methods originated in the 1960s^34,35^ and were developed to track single or a hand-full of objects with complex distinguishing features such as ships or airplanes. However, here we require the tracking of objects that are visually almost identical. In some cases this can be resolved by maintaining multiple association hypotheses over multiple time points^36^. However, although particle trackers and state space models can produce high-quality results, proofreading (data curation) is always required in order to guarantee error-free tracks. Notably, computer-assisted approaches for proofreading are usually not related to the method that produced the automated results in the first place.

Interactive error correction is rarely part of available tracking systems and usually turns out to be difficult to implement and integrate, leaving the user with an inflexible patchwork of multiple tools. Part of the reason for this is the way classical tracking models work. Their local and iterative solvers are highly specialized, not offering intrinsic possibilities to constrain the space of possible solutions in a user-driven way. In other words, they intrinsically do not offer any interaction capabilities that can be employed by users to prevent the tracking system from making certain mistakes.

Assignment Models promise to make a difference in all these respects. The novelty of this type of tracking system is the way in which solutions are found. A tracking problem is formulated as a global optimization problem under constraints that can be solved using discrete optimization methods. MoMA is based on such an optimization-based assignment model that allows the user to furnish constraints in an interactive manner. Hence, we can offer unprecedented user interactions for data curation—a process we call leveraged editing.

In particular, MoMA offers the following leveraged editing primitives: (i) Forcing solutions to contain a selected cell (segment), (ii) forcing solutions not to include specific segments, (iii) forcing a cell to a given movement or division (assignment), or to (iv) avoid such an assignment, and (v) specifying the number of cells visible at a given time. We will show that the very nature of the underlying optimization problem allows us to seamlessly incorporate these leveraged editing primitives.

### Automated tracking with MoMA

Here we briefly review the class of tracking methods called assignment models^23,37–40^. We provide sufficient technical detail to prepare the reader for later sections, introducing leveraged editing primitives used in MoMA.

Tracking consists of two equally important tasks: Cells need to be segmented in each frame, and segments of the same cell in consecutive frames need to be linked. Tracking by assignment approaches these tasks by formulating and solving a joint global optimization problem. In this context, the segmentation task consists of selecting a subset of segments in each image, i.e. corresponding to the cells in the image. To do this, the algorithm first generates a large collection of possible segment hypotheses that are contained in a (possibly heavy) oversegmentation of the images. Joint segmentation and tracking then boils down to enumerating many potential subsets of segments together with potential ways of linking (assigning) these between consecutive frames. To identify, among all these possible joint segment/assignment subsets, an optimal solution, each of the potential segments and assignments is given a cost. The cost of a joint segmentation/assignment hypothesis aims to reflect how unlikely it is that the corresponding dynamics occurs in the real system, i.e. the corresponding movement, growth, and division of the cells in our system. That is, the total cost can be thought of as a negative log-likelihood of the segmentation/assignment hypothesis^38,41^ and an optimal solution minimizes this cost.

The cost function is designed to reflect the knowledge of domain experts. To give an example, in our application, the cost function for a cell division assignment that links one segment to two segments in the next frame contains a term that evaluates the size of the three segments to be linked which implements the physical constraint that the sum of the sizes of the two daughter cells should be similar to that of the mother cell. Structural knowledge about which assignments can be chosen simultaneously is encoded in terms of constraints that ensure that only physically meaningful solutions can be chosen. That is, solutions that do not describe impossible events like cells popping into existence out of nowhere, cells moving to two places at once, etc. In our implementation, these constraints force or prohibit certain segments and assignments to be jointly contained in a segmentation/assignment solution. Notably, in formulating these constraints we of course take advantage of the fact that the microfluidic device organizes cells into one-dimensional arrays.

Once the segmentation and tracking problem has been formalized in this manner in terms of costs and constraints, well-established discrete optimization methods can be used to obtain a solution that is (i) feasible, i.e., free of conflicts, and (ii) cost-minimal. In the following we will put these notions on formal grounds. A more in-depth description can be found in ref. ^23^, where we described in detail the assignment model upon which MoMA operates. In the next section we will briefly summarize this model in order to lay the foundation to understand the leveraged editing primitives introduced thereafter.

### The assignment tracking system in MoMA

First, an excess of segment hypotheses *H*^(*t*)^ is generated for each frame *t*, with many hypotheses partially overlapping and thereby providing alternative and mutually exclusive interpretations of where the cells are appearing in the image^23^. To represent possible solutions, a binary segmentation variable *h*^(*t*)^ is associated to each possible segment hypothesis in *H*^(*t*)^. Whenever *h*^(*t*)^ = 1, it indicates that this segment hypothesis is part of the proposed solution. Similarly, a set of assignment hypotheses *A*^(*t*)^ and associated binary assignment variables *a*^(*t*)^ are generated, that link segment hypotheses at time point *t* to segment hypotheses at *t*+1. For example, a mapping assignment $a_{i \mapsto j}^{(t)}$ connects two segment hypotheses $h_i^{(t)}$ and $h_j^{(t + 1)}$.

Thus, a proposed segment/assignment solution consists of a selection of binary segmentation and assignment variables *v* that are set to 1. As mentioned above, a cost function is defined that associates to every such variable *v*, a cost $c_v \in {\Bbb R}$ of including it in a solution. For details on the cost function used for mother machine devices, we refer to ref. ^23^. In a nutshell, the cost measures how much a segment/assignment deviates from the expected appearance/dynamic behavior of bacterial cells. The total cost *C* of a particular solution is then simply the summed cost over all active variables

1 $$C = \mathop {\sum}\limits_i v_i \cdot c_{v_i}.$$

Linear constraints are used to restrict the solution space to only include conflict-free and structurally sound solutions. As a simple example, two segment hypotheses that offer conflicting explanations of a particular pixel cannot simultaneously be active in any feasible solution. To introduce some notation that will be required below, we look in detail at one particular constraint. Continuity constraints ensure that each active segment at frame *t* (i.e. each cell) must be involved in exactly one assignment entering from frame *t*−1 and must also be involved in exactly one assignment towards *t*+1. In other words, each cell must have a past and a future. Formally, this statement can be written as

2 $$\forall t \in \{ 2, \ldots ,T - 1\} ,\forall h^{(t)} \in H^{(t)}:\mathop {\sum}\limits_{a^{(t - 1)} \in {{\Gamma }}_{\mathrm{L}}\left( {h^{(t)}} \right)} a^{(t - 1)} = h^{(t)} = \mathop {\sum}\limits_{a^{(t)} \in {{\Gamma }}_{\mathrm{R}}\left( {h^{(t)}} \right)} a^{(t)}.$$

Here we image time frames ordered from left to right and use the notation *Γ*_L_(*h*) to denote the set of assignments directly to the left of segmentation variable *h* (i.e. its left neighborhood) and *Γ*_R_(*h*) to denote the set of assignments directly to the right of *h* (its right neighborhood). That is, the left neighborhood *Γ*_L_(*h*) is the set of all assignments entering *h f*rom the previous frame and the right neighborhood *Γ*_R_(*h*) is the set of all assignments leaving *h* towards the next frame. The equation above then says that, for each cell at time *t*, there should be one assignment in the previous time frame, and one in the following time frame.

A globally optimal solution, i.e. picking a set of conflict-free assignments of minimal cost can be achieved by solving an integer linear program (ILP)^23,38–40^.

An ILP is an optimization problem that is fully specified by (i) an objective function that is a linear function of a set of variables ${\cal V}$, and (ii) a set of constraints that are formalized as (in-)equalities on these variables. The space of feasible solutions is defined by all variable assignments that obey all constraints. An optimal solution is a feasible solution that minimizes the objective function.

The joint segmentation and tracking formulation introduced above is already in ILP form: The set of variables ${\cal V}$ comprises binary segmentation and assignment variables. The objective to minimize is the cost *C* defined in Eq. (1). Note that this is a linear function of the variables $v \in {\cal V}$. In Eq. (2) we also gave an example of how constraints can be formalized as linear equalities.

Integer linear programming is a well-understood problem^42^, and given the above formulation we can turn to off-the-shelf solvers like Gurobi to find an optimal solution. Although finding an optimal ILP solution is NP-hard, recent success solving relatively large tracking problems^23,38–40^ suggests that assignment models pose well-natured instances to be solved as ILPs.

In the following we will make use of a particular feature of many ILP solvers, namely the ability to perform “warm-starts”. One speaks about a warm start if a solver can benefit from residual intermediate results created during a preceding optimization. This can speed-up optimization significantly as shown in ref. ^23^.

Additional performance for solving the ILP underlying a tracking instance can be gained by reducing variable redundancy via substitution. The set of variables ${\cal V}$ contains variables *h* for available segments, and *a*, for available assignments. However, note that whenever the segmentation variable for a segment *i* is active, i.e. *h*_*i*_ = 1, then at least one assignment *a* that involves a segment *i* must be active as well. Using these constraints, the segmentation variables can be removed from the model entirely^23^. That is, after adequate constraints are added to the ILP, each occurrence of $h_i^{(t)}$ can be substituted by a sum over all assignment variables in *Γ*_L_$\left( {h_i^{(t)}} \right)$ (or *Γ*_R_$\left( {h_i^{(t)}} \right)$).

### Leveraged editing of tracking solutions

In this section we discuss how MoMA modifies the underlying optimization problem in response to user feedback. MoMA first of all provides the user with a graphical interface that allows the user to browse through the tracking solution that the optimization has provided for a given movie. The basic idea of leveraged editing is simple: When a user identifies a segmentation or tracking error, (s)he suggests the correct alternative or simply points at the error in the graphical interface, leaving the algorithm to search for a corrected solution to the model. In MoMA, the given feedback is incorporated into the ILP via additional constraints. Using warm-starts allows optimizing the modified problem fast enough for interactive use. Fixing a single error will usually resolve a bulk of transitive errors. These interaction-based modifications and re-optimizations are iterated until the found solution is satisfactory to the user, i.e., appears to be free of errors.

Here we introduce five specific interaction primitives implemented in MoMA. We will see that they do not introduce significant changes to the existing assignment tracking formulation and can be implemented efficiently. To illustrate how leveraged editing works in practice, a tutorial movie is available on MoMA’s Wiki page^30^, showing several of these primitives in action.

One possible error is that the tracking may have failed to include a particular cell, possibly even across multiple frames. In this case, the user wants to choose an adequate segment and force it to be included in the tracking solution. In MoMA this can be achieved by hovering the mouse over the part of the image where a cell was not picked up by the original optimization. Segment hypotheses located at the mouse position will be highlighted interactively, and simply clicking on any highlighted segment will cause (i) adequate modifications of the ILP (as described below), and (ii) a re-run of the solver to obtain an optimal solution for the given data, now constrained to include the forced segment.

Technically this can be achieved by adding a single constraint to the ILP, namely *h*_i_ = 1 where *h*_*i*_ is the chosen segment. Applying the redundancy reduction discussed in the previous section, the constraint to be added can be expressed in terms of assignment variables as

3 $$\mathop {\sum}\limits_{a \in {{\Gamma }}_{\mathrm{R}}\left( h \right)} a = 1,$$

where *Γ*_R_(*h*) is the right neighborhood of *h*, i.e. the set of all assignments leaving *h* towards the next frame.

In addition to allowing users to force missing segments to be included, the user can also tell MoMA to exclude certain segments from solutions. The re-solved ILP will correspond to the minimal cost solution for the data, constraint to exclude the chosen segment. Analogously to forcing segments, the constraint to be added to the ILP is

4 $$\mathop {\sum}\limits_{a \in {{\Gamma }}_{\mathrm{R}}\left( h \right)} a = 0.$$

Instead of interacting with segments, a user might want to directly work with individual assignments. To do so, users can browse through a library of available assignments. Assignments can be included or excluded from tracking solutions.

Browsing the library of available assignments can be done in only a few mouse-clicks. Since there is precisely one binary variable *a* corresponding to the chosen assignment, the constraint to be added to the ILP to force or exclude this assignment is simply *a* = 1 and *a* = 0, respectively.

The last interaction primitive of MoMA is particularly powerful, often capable of fixing multiple tracking errors at once. The idea is simply to let MoMA know how many cells are contained in a given time point. We constrain the solution space to only allow solutions that contain *k* segmented cells at time point *t*. Formally this is accomplished by adding the constraint

5 $$\mathop {\sum}\limits_{h \in H^{(t)}} \mathop {\sum}\limits_{v \in {{\Gamma }}_{\mathrm{R}}(h)} v = k,$$

where *H*^(*t*)^ is the set of all segments existing at time *t*.

### Installation of MoMA

The installation of MoMA can be performed via Fiji^43,44^. In Fiji, simply activate the MoMA update site. Once installed, the Fiji updater will automatically install future versions of MoMA containing new features and bugfixes. The MoMA Wiki pages contain further information about how to install and use MoMA^30^.

### Implementation of MoMA

MoMA is implemented in Java, using the imaging library ImgLib2^45^ and other components from the open source universe around ImageJ and Fiji^43,44^. For solving ILPs we use Gurobi. The source code of MoMA is a Maven project, hosted on GitHub^30^.

### Additional features of MoMA

Additional useful features of MoMA include (i) the ability to optimize (solve) only parts of a loaded data set, (ii) save a fully or partially curated data set, and (iii) the possibility to export a found tracking solution for downstream processing.

If a loaded data set contains 1000 or even more time points, the optimization of MoMA’s assignment model can take tens of seconds. While this is still fast, e.g. when compared to the data acquisition time for such a data set, leveraged editing can become cumbersome when the user is forced to wait tens of seconds between interactions for the optimization to finish. In order to guarantee fast interactive response times, MoMA allows users to define a subrange of time points [*t*_*a*_, *t*_*b*_] across which to perform the optimization.

All assignments that are not in [*t*_*a*_, *t*_*b*_] are either set to the value computed at a previous (partial) optimization run, or simply clamped to be 0. Formally this can be expressed by

6 $$\forall t \,\notin \,\left[ {t_a, \ldots ,t_b} \right],\forall a^{(t)} \in A^{(t)}:a^{(t)} = \left[ {\begin{array}{*{20}{l}} {1} \hfill & {{\mathrm{if}}\,a^{(t)}\,{\mathrm{was}}\,{\mathrm{set}}\,{\mathrm{to}}\,{\mathrm{1}}\,{\mathrm{previously}},{\mathrm{or}}} \hfill \\ {0} \hfill & {{\mathrm{otherwise}}{\mathrm{.}}} \hfill \end{array}} \right.$$

Once correct solutions are found, it is important that users can save and load the curations that they performed. Leveraged editing primitive introduces additional constraint to the underlying optimization problem, and MoMA is capable of serializing all edits to file.

But not only leveraged edits can be saved, also MoMA’s segmentation and tracking results can be exported for subsequent downstream processing. An exhaustive list of exportable data is given below. MoMA’s Wiki page contains a formal specification of the used data format^30^.

- Data source.
- Total number of cells observed in the data set.
- Number of channels in the raw data, i.e. phase contrast and fluorescent channels.
- Growth channel (growth-channel) height and image height in pixels.
- (Vertical) position of the growth-channel in the image.
- For each cell, its cell id, and lineage information (the ids of its ancestors).
- For each time point in the life of each cell: position in the growth-channel [pixels and cell number]; bounding box area; intensity histogram, intensity percentiles, and pixel intensities for all channels.

### Curation statistics

MoMA was tested on Mother Machine data with ~30 frames per cell cycle, stable focus over the experiment and both phase contrast and fluorescence imaged. To estimate the time the user needs to spend to curate data sets, we analyzed an unbiased selection of growth-channels and measured the time spend curating. For the selection of the growth-channels there was no visual inspection of the growth-channels other than checking that they harbor cells on the first frame. Therefore, this sample also harbored growth-channels in which the cells are lost during the experiment, and some that show structural defects. We only used the times for the growth-channels in which we had cells until the end of the experiment. Defect growth-channels were excluded as well. There are also rare growth-channels in which a cell is lysing or shows other abnormalities. In such cases, even with the eyes of an experienced observer, it is difficult to decide on the border of such cells, and such growth-channels were excluded as well.

Figure 1f shows a histogram of the fraction of frames needing curation across the growth-channels. Roughly half of the growth-channels required no curation at all, and most growth-channels require less than 1% of frames curated, with about 3% of frames needing curation in the worst case.

**Fig. 5:**
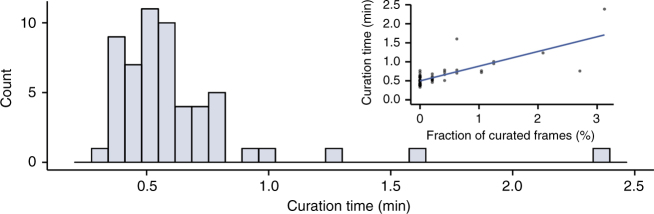
Histogram of the curation times per 100 frames for a representative set of growth-channels. The inset shows a scatter plot of curation times (per 100 frames) as a function of the fraction of frames that were curated. The line shows a linear regression (least squares) fit

To give an impression of the amount of time that these curation statistics correspond to, in our hands, Fig. 5 shows the distribution of curation times per 100 frames across the growth-channels we analyzed. For each growth-channel, the total number of curated frames was extracted from the serialized file of curation interactions that MoMA saves. The inset of Fig. 5 shows that curation times are generally correlated with the fraction of frames that required curation.

All the curating with MoMA was performed on a MacBookPro (2.4 GHz Intel Core i7, 8 GB of memory). On this setup loading, initialization and the first round of optimization of a data set with 480 frames with two channels typically takes around 1 min. After curating the data the export step takes another 30 s.

### Cell size and growth rate estimation

From the imaging data we obtain, for each cell, pictures for each time point during its life-cycle. As an estimate of cell size, the software provides the dimension of the rectangular bounding-box within which the cell is contained. We have found that, both on our own data as on the data from other devices and microscopy setups, virtually all cells accurately follow simple exponential growth curves as a function of time, supporting the robustness of the estimation procedure. However, it is clear that the cell size estimation is quite coarse and we aimed to quantify the accuracy of these size estimates. This is difficult to do directly because we do not have independent measurements of cell sizes that can be used as a gold standard. However, if we find that the estimated cell size *s*(*t*) accurately follows a simple exponential or linear form as a function of time *t*, then this suggests the errors in cell size are at most as large as the fluctuations of *s*(*t*) away from the simple exponential or linear growth law.

**Fig. 6:**
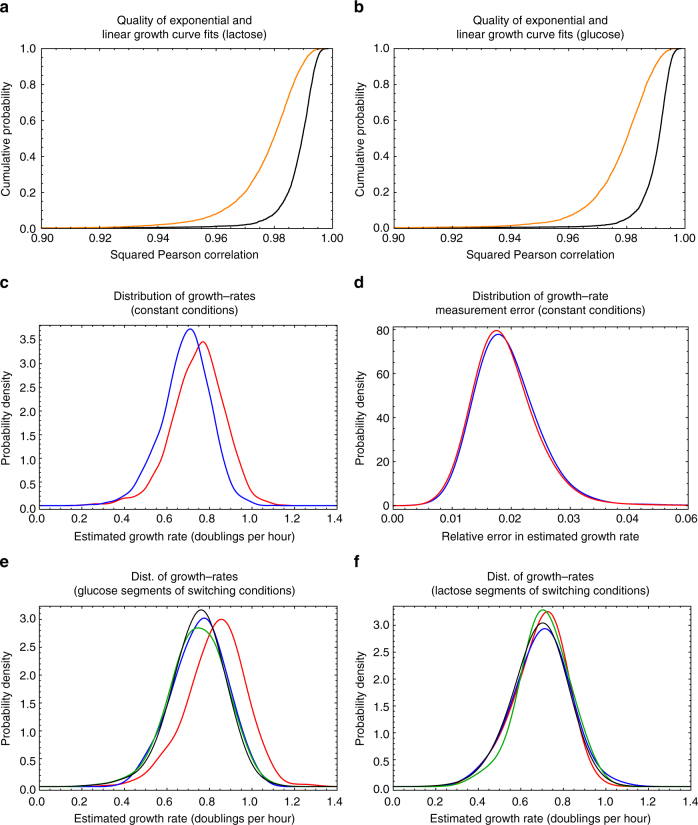
Exponential growth curves and the distribution of growth rates. **a** Cumulative distributions of the squared Pearson correlations between the estimated sizes of the cells and time (orange), or estimated log-sizes and time (black) for the cells grown in lactose. **b** As in panel **a** but for cells grown in glucose. **c** Distribution of growth rates of individual cell cycles in glucose (blue) and lactose (red). **d** Distribution of relative errors of the growth rate estimates in glucose (blue) and lactose (red). **e** Distribution of growth rates of individual cell cycles during the first (red), second (blue), and third (green) time segments in glucose of the experiments with switching conditions. For comparison, the black curve shows the growth rate distribution in constant glucose. **f** As in panel **e** but for the time segments in lactose

Let *s*(*t*) be the estimated size of the cell at time *t* and $x(t) = {\mathrm{log}}[s(t)]$. We used the data sets from the constant environments and used all cells which were monitored from birth to division, corresponding to 4016 cell cycles in glucose and 3387 cell cycles in lactose. For each cell cycle, we calculated the Pearson correlation between *x*(*t*) and *t* across the cell cycle, as well as the Pearson correlation between *s*(*t*) and *t*. Figure 6 shows the cumulative distributions of the squared Pearson correlation of the growth curves with exponential (black) and linear (orange) functions for cells grown in lactose (Fig. 6a) and in glucose (Fig. 6b).

We see that the growth curves are very well described by exponential functions of time, i.e. the median squared correlation coefficient is approximately 0.99 and almost all cells have correlation coefficients larger than 0.98. Correlation coefficients are substantially lower for fits to linear growth curves. Note that, whereas correlation coefficients are still very high for the linear growth fits, the log-likelihood for a growth-curve with squared correlation *r*^2^ and *T* time points scales as $- T\,{\mathrm{log}}[1 - r^2]$. Thus, for a typical cell-cycle with *T* = 30 time points, the likelihood ratio between a fit with *r*^2^ = 0.99 and one with *r*^2^ = 0.98 is ${\mathrm{exp}}(20.8) \approx 10^9$. That is, the differences in the qualities of the linear and exponential fits are highly significant.

Since the elongation of cells is very well described by an exponential model, we can estimate the measurement error by studying the residuals of these fits. These residuals represent an upper bound on length measurement errors since they also include biological fluctuations around constant exponential growth. For each cell size observation in each cell cycle, we calculate the squared residual from the exponential fit, and obtained a squared relative error by dividing by the square of the estimated size. We then stratified the errors according to size and calculated, for each size class, the means and standard deviations of the squared relative errors. Taking the square-roots of these values we finally obtain the relative errors of the size measurements as a function of estimated size (Fig. 1g). We find that the measurement error on size is between 2 and 3%, and approximately independent of the length itself.

To estimate the average growth rate of an individual cell cycle we use linear regression of the log-sizes $x(t) = {\mathrm{log}}[s(t)]$ across the time points *t* in the cell-cycle, i.e. assuming all deviations from a perfect linear relationship *x*(*t*) = *a*(*t*−*t*_0_)+*x*_0_ are due to errors in the log-size estimates *x*(*t*). Marginalizing over the cell-size *x*_0_ at the time of birth *t*_0_, we find that the standard-deviation of the posterior distribution over growth-rate *a* is given by

7 $$\sigma (a) = \sqrt {\frac{{{\mathrm{var}}(x)(1 - r^2)}}{{(T - 1){\mathrm{var}}(t)}}} ,$$

where var(*x*) and var(*t*) are the variances of the log-sizes *x*(*t*) and measurement times *t*, *T* is the number of measurements in the cell cycle, and *r* is the Pearson-correlation of the linear fit. The relative error on the estimated slope a_*_ = cov(*x*, *t*)/var(*t*) is given by the ratio *σ*(*a*)/a_*_.

Figure 6c shows the distribution of growth rates that we observe in constant glucose and lactose, and Fig. 6d shows the distribution of relative errors on growth rate. For the large majority of cell cycles, the error on the estimate of the growth rate is between 1 and 3%. The average growth rate is a bit higher in glucose (0.75 doublings per hour) than in lactose (0.69 doublings per hour). Notably, the variation in the growth rates of individual cell cycles is much larger than the measurement errors on these growth rates, indicating that growth rates vary considerably across single cells. We find that growth rates vary by about 17% in both glucose and lactose (i.e. one standard deviation), and we observe cell cycles that differ by more than twofold in their growth rates.

We also investigated whether growth rates during the switching conditions vary systematically from growth rates in the corresponding constant conditions. Figure 6 shows the distribution of growth rates for individual cell cycle separately for the first, second, and third time segment in both glucose (Fig. 6e) and lactose (Fig. 6f) during the switching conditions.

We see that the growth rate distributions during individual time segments in the switching experiments are very similar to the distributions in the corresponding constant conditions. The only exception is the slightly higher growth rates in the first time segment in glucose during the switching conditions. Although we have not investigated the origin of the slightly higher growth rates in this time segment in detail, we believe that it results from a combination of two effects. First, we note that the growth rates in glucose are slightly higher in all three segments during the switching conditions than in the constant conditions. This suggests that a subtle change in the conditions on the day of the experiment may have caused slightly increased growth rates during the switching conditions. Second, when fluorescence measurements are taken, the light from the illumination causes some small stress to the cells, which is reflected in slightly lower growth rates compared to conditions where no fluorescence measurements are taken. As a consequence, we observe that cells slightly lower their growth rates during the first hours of the experiment. To correct for this systematic effect we only start recording measurements in each experiment, after 2 h in conditions with illumination. We hypothesize that during the first glucose segment in the switching experiments, the cells had not yet fully adapted to the illumination conditions.

### Cell fluorescence estimation

**Fig. 7:**
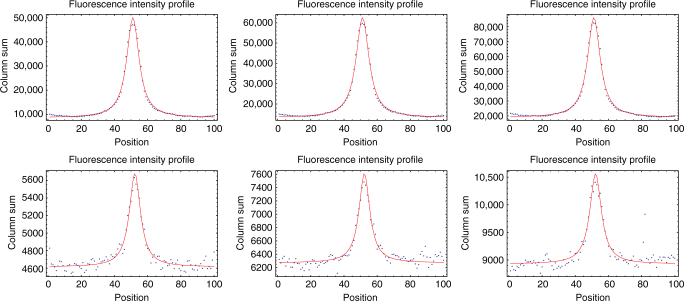
Examples of the horizontal profiles of column sums *c*_*i*_ of fluorescence intensities (blue dots) for three cells during growth on lactose (top panels) and during growth on glucose (bottom panels). The three panels show, from left to right, the profiles at the start, the middle, and at the end of the cell cycle of two example cells. The red curves show the fits to the mixture model of a fixed background plus a Cauchy-distributed signal

To estimate the GFP content of each cell, we post-process the fluorescence data as follows. The raw data consist of fluorescence intensities for all pixels within the segment of the picture containing the cell. This segment is 100 pixels wide, with the growth-channel covering approximately 13 pixels in the center of the picture. We first obtain column-sums *c*_*i*_ by summing the pixel intensities of all pixels in each of the 100 columns *i*. Note that we assume that these column sums are dominated by the fluorescence coming from the cell in question, i.e. that the fluorescence coming from neighboring cells above and below the cell are negligible. We find that this is a good approximation when cells in a given growth-channel all have similar fluorescences but note that, in conditions where neighboring cells may have fluorescences that differ by orders of magnitude, this assumption may break down. Figure 7 shows the profiles of column sums *c*_*i*_ for a cell at three time points of its cell cycle while growing in lactose (top three panels) and for a cell growing in glucose (bottom three panels). From prior biological knowledge, we know that GFP is highly expressed during the growth on lactose, and that it is very lowly expressed during the growth on glucose.

Remarkably, the growth-channel (central 13 pixel positions in the figures) is not detectable at all in the fluorescence curves. Instead, the fluorescence signal shows a long-tailed peak centered in the middle of the growth-channel, extending far beyond the width of the growth-channel, and reaching a minimum at positions halfway between neighboring growth-channels, i.e. near the left and right ends of the profiles in Fig. 7. As the cell grows, i.e. from the leftmost to rightmost panel, the length of the segment grows and the column-sums grow proportionally to the segment length. Notably, the minimal fluorescence level is almost twice as high when growing in lactose compared to when growing in glucose. We conclude from these observations that the fluorescence from each cell spreads over significant distances across the image and that this also causes background levels to depend on the overall expression levels in neighboring growth-channels. Therefore, to properly estimate the amount of fluorescence emerging from the cell we need to fit the background intensity within each segment and we need a mathematical model for the long-tailed shape of the peak.

We found that the shape of the peak is very well described by a Cauchy (or Lorentzian) distribution, giving an overall form of the fluorescence profile:

8 $$c_i = {\mathrm{noise}} + B + \frac{A}{{1 + \left( {\frac{{i - i_{{\mathrm{mid}}}}}{w}} \right)^2}},$$

where *i* is the horizontal position, *i*_mid_ is the center of the peak, *w* its width, *A* the amplitude of the signal, ‘noise’ is the measurement noise, and *B* the background fluorescence. Assuming that the measurement noise is Gaussian distributed, it is straight forward to fit this model using expectation maximization. We find that, systematically, the center *i*_mid_ ≈ 50–52, and the width *w* ≈ 5–6 pixels. We interpret the amplitude *A* to be proportional to the total number of GFP molecules in the cell, and the background *B* to correspond to the combined effects of the camera offset, the auto-fluorescence of the microfluidic chip and the media, and stray fluorescence from neighboring cells. The expectation maximization procedure for fitting the fluorescence profile is

1. Find the maximum and minimal fluorescence column-sums *c*_max_ and *c*_min_ across the profile.
2. Initialize *B* to *c*_min_, *A* to *c*_max_−*c*_min_, *w* to 5.5 and *i*_mid_ to 50, i.e. in the middle of the profile.
3. Calculate a theoretical profile:
  9 $$\rho _i = \left[ {1 + \left( {\frac{{i - i_{{\mathrm{mid}}}}}{w}} \right)^2} \right]^{ - 1},$$
  and its integral $\rho = \mathop {\sum}\nolimits_{i = 1}^N \rho _i$.
4. Set a new value of the background *B*′:
  10 $$B^{\prime} = \frac{1}{N}\mathop {\sum}\limits_{i = 1}^N \frac{{Bc_i}}{{B + A\rho _i}}.$$
5. Set a new value for the amplitude *A*′:
  11 $$A^{\prime} = \frac{1}{\rho }\mathop {\sum}\limits_{i = 1}^N \frac{{Ac_i\rho _i}}{{B + A\rho _i}}.$$
6. Using the updated values *A*′ and *B*′, calculate an updated profile *ρ*_*i*_ and optimize *i*_mid_ by finding the zero of the derivative:
  12 $$\mathop {\sum}\limits_{i = 1}^N \left( {i - i_{{\mathrm{mid}}}} \right)\rho _i^2\left( { - 1 + \frac{{c_i}}{{B^{\prime} + A^{\prime}\rho _i}}} \right).$$
7. Using the updated values *A*′, *B*′, and *i*_mid_, optimize *w* by finding the zero of the derivative:

13 $$\mathop {\sum}\limits_{i = 1}^N \rho _i(1 - \rho _i)\left( { - 1 + \frac{{c_i}}{{B^{\prime} + A^{\prime}\rho _i}}} \right).$$

The accuracy of this method to estimate the total fluorescence of the cell can be quantified by taking advantage of the precise environment control allowed by our new setup, as discussed in the next section. We distribute a post-processing script with the MoMA code that allows users to apply this fluorescence amplitude estimation to exported output files from MoMA.

### Cell auto-fluorescence estimation

In addition to the background fluorescence of the PDMS and stray fluorescence from nearby cells that are corrected for by the methods described in the previous section, there is background fluorescence coming from the auto-fluorescence of the cells and media. To estimate this auto-fluorescence, we measured the wild-type strain of *E. coli* MG1655, i.e. without the fluorescent reporter, in the conditions where we switch between glucose and lactose. We observed that the estimated total fluorescence, i.e. the amplitude *A* from the previous section, correlates well with the size of the cells during their cell cycle. That is, fitting a linear relationship *A* = *aS*+*b* of the fluorescence *A* as a function of the estimated cell size *S* typically yields Pearson correlation coefficients of *r* ≈ 0.9. Moreover, we observed that the vast majority of fits were consistent with *b* = 0, i.e. the total fluorescence being directly proportional to cell size, supporting that this signal corresponds to the auto-fluorescence of the cell. Note that any uniform fluorescence coming from the growth medium would also be accounted for by this procedure (in the parameter *a*).

To fit the auto-fluorescence *a* (per micrometre of cell length) we selected all cells that were observed for a full cell cycle, who never got within 100 pixels of the end of the growth-channel during their cell cycle, and whose length as a function of time was well fit by a simple exponential growth curve (*r*^2^ ≥ 0.99). This latter restriction mainly serves to remove cells that had a transient stop in growth after the first switch to lactose. In total there were 284 cells that passed all these criteria. For each of these cells we replaced the directly estimated sizes *S*_*t*_ at each time point *t*, with the sizes $\tilde S_t$ estimated from the exponential fit of *S*_*t*_ as a function of time (reasoning that these estimates are more accurate than the direct measurements). For each cell we then fit a function $A_t = a\tilde S_t$, assuming Gaussian measurement noise of unknown variance.

That is, for a single cell we write

14 $$P\left( {D|a,\sigma } \right) \propto \sigma ^{ - T}\,{\mathrm{exp}}\left[ { - \mathop {\sum}\limits_t \frac{{\left( {A_t - a\tilde S_t} \right)^2}}{{2\sigma ^2}}} \right].$$

Using a scale prior on *σ* of the form *P*(*σ*) ∝ 1/*σ*, and integrating over *σ* we obtain

15 $$P(a|D) \propto \left[ {\left\langle {\tilde S^2} \right\rangle \left( {a - \frac{{\left\langle {A\tilde S} \right\rangle }}{{\left\langle {\tilde S^2} \right\rangle }}} \right)^2 + \left\langle {A^2} \right\rangle - \frac{{\left\langle {A\tilde S} \right\rangle }}{{\left\langle {\tilde S^2} \right\rangle }}} \right]^{ - T/2},$$

where *T* is the number of time points in the cell cycle and the averages are over the time points in the cell cycle.

The optimal value of *a* is given by

16 $$a_ \ast = \frac{{\left\langle {A\tilde S} \right\rangle }}{{\left\langle {\tilde S^2} \right\rangle }}.$$

Approximating the posterior by a Gaussian we obtain for the standard deviation of the estimated *a*

17 $$\sigma _a = \sqrt {\frac{1}{T}\left[ {\frac{{\left\langle {A^2} \right\rangle }}{{\left\langle {\tilde S^2} \right\rangle }} - a_ \ast ^2} \right]} .$$

**Fig. 8:**
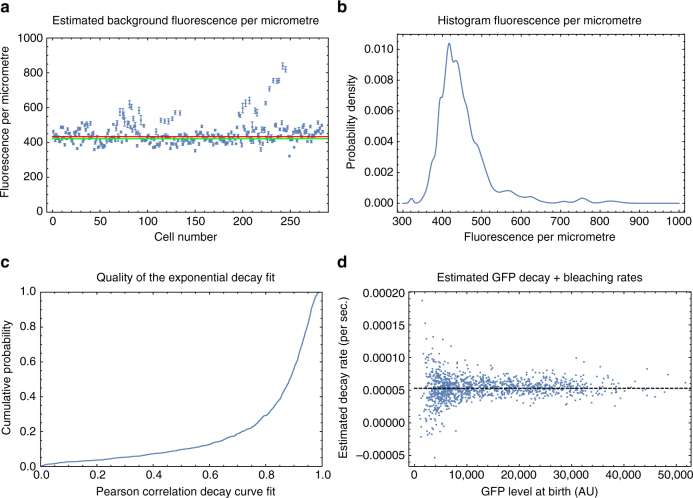
Estimating auto-fluorescence and fluorescence decay. **a** Estimated auto-fluorescence per micrometre cell length a_*_ and its error bar *σ*_*a*_ for 284 cells for which fluorescence was fit as a function of cell size. The red line (*a* = 433.5) is the fit obtained when all cells are assumed to have a common fluorescence per micrometre *a*. The green line is obtained with a mixture model that allows for “outliers” from a uniform distribution (*a* = 421.8). **b** The joint probability density of *a* given by the mixture of Gaussian distributions for all 284 cells. **c** GFP decay (including bleaching) was estimated by fitting observations of decreasing GFP levels during the glucose phases in the switching experiments, when no GFP is synthesized, to an exponential function of time. The panel shows the cumulative distribution of the Pearson correlations between the estimated log-GFP levels and time. **d** Estimated decay rates for individual cells (vertical axis) as a function of absolute GFP level of the cell (horizontal axis). The dashed line shows the overall estimated rate used in subsequent analysis

Figure 8a shows the estimated value a_*_ and its error bar *σ*_*a*_ for each of the 284 cells. Note that, although most cells have fluorescence values between 400 and 500 per μm, there are some outliers at higher fluorescence. This is also evident from the combined probability density of *a* values (Fig. 8b).

If we assume there is one common background fluorescence per micrometre *α* for all cells, then the probability of the data given *α* is given by

18 $$P(D|\alpha ) = \mathop {\prod}\limits_c \frac{1}{{\sigma _a(c)}}{\mathrm{exp}}\left[ { - \frac{{\left( {a_ \ast (c) - \alpha } \right)^2}}{{2\sigma _a(c)^2}}} \right],$$

where the product is over the 284 cells *c*.

Maximizing this function with respect to *α* yields

19 $$\alpha = \mathop {\sum}\limits_c \frac{{a_ \ast (c)}}{{\sigma _a(c)^2}}\left[ {\mathop {\sum}\limits_c \frac{1}{{\sigma _a(c)^2}}} \right]^{ - 1} = 433.5.$$

If we allow that there are some ‘outlier’ cells whose value of *a* is described by a uniform distribution of width *W* = *a*_max_ − *a*_min_, then the likelihood of the data as a function of *α* and the fraction of non-outlier measurements *ρ* is given by

20 $$P(D|\alpha ,\rho ) = \mathop {\prod}\limits_c \left[ {\frac{{1 - \rho }}{W} + \frac{{\rho \,{\mathrm{exp}}\left( { - \frac{{\left( {\alpha - a_ \ast (c)} \right)^2}}{{2\sigma _a(c)^2}}} \right)}}{{\sqrt {2\pi } \sigma _a(c)}}} \right].$$

Maximizing this function with respect to *α* and *ρ* yields *α* = 421.8 and *ρ* = 0.31. In the following we will use this latter value of *α* for the auto-fluorescence per micrometre of cell length. For each cell with estimated size *S* and total fluorescence *A*, we thus obtain an auto-fluorescence corrected fluorescence level $\tilde A = A - \alpha S$.

### Estimating GFP’s bleaching and degradation

As shown in Fig. 2a, while the *lac* operon is induced in the lactose phases, GFP production ceases during the glucose phases. In this regime, the total cell fluorescence slowly decreases during the cell cycle, and approximately divides in half at each cell division. We reasoned that the slow continuous decay of fluorescence during the cell cycles is the result of GFP bleaching and, potentially, also some GFP degradation. Inspection of the data indeed shows that the total fluorescence decrease is captured well by an exponential model. For this analysis, we consider only observations between 30 min after the switch to glucose and before the next switch to lactose, and to cells with at least 10 points in this time window. This corresponded to 33,052 independent cell observations over 1220 cells.

As shown in Fig. 8c, the GFP degradation across time is well fit by an exponential model for most cells. Assuming that a cell undergoes bleaching+GFP degradation at a rate *μ* per second, we estimated *μ* for each cell from a linear regression of log(GFP level) against time (Fig. 8d). Combining information from the estimates of individual rates for each cell and their standard deviations, we estimate the overall rate μ_*_ to be equal to 5.3×10^−5^ ± 5×10^−7^ (mean ± s.d.) per second. Note that this corresponds to a loss of about 18% of the GFP signal per hour due to bleaching and GFP decay.

### Accuracy of the fluorescence estimation

We also took advantage of our unique ability to study the growth regime where no GFP is produced to quantify the measurement errors on the total GFP. Since, in the glucose phases of the switching experiments, the GFP dynamics is dominated by bleaching and degradation, and well described by an exponential decay model, we computed the squared residuals (normalized by the squared value) from the independent fits of log(GFP) as a function of time for each cell. As for the analysis of measurement errors on length, residuals are stratified into bins based on total GFP, and the means and standard errors of the normalized squared residuals (i.e. relative to total GFP) are computed for each bin (Fig. 2g). We find that the squared relative error on the GFP measurement scales inversely with the total GFP level (i.e. a power-law fit has exponent 1.01), which indicates that, as in shot noise, the squared error is inversely proportional to total GFP level. In practice, the absolute error is around 20 molecules when the cell has 200 GFP molecules (i.e. 10%), and around 80 molecules when the total is 4000 GFP molecules (i.e. 2%).

### Estimating the fluorescence per GFP molecule

To estimate the conversion factor between the background-corrected total fluorescence $\tilde A$ and the number of GFP molecules, we will use data on the fluctuations in fluorescence levels of newborn sibling pairs. To avoid confounding effects from GFP production, we collected division events from the glucose phases in our switching experiments, when GFP production has ceased. Collecting division events from these phases has the added advantage that absolute GFP levels vary over a considerable range across cells during these phases, allowing us to quantify the size of fluctuations in sibling fluorescence as a function of total fluorescence. Our observations consist of fluorescence levels at birth (*x*_*i*_, *y*_*i*_) for sibling pairs of daughters, where *i* runs from 1 to *N*, with *N* the total number of such sibling pairs. Using the same criteria as in the decay analysis for mothers and daughters, we collected *N* = 357 sibling pairs. GFP levels at birth were estimated in each daughter cell as average of the levels at all time points corrected for the previously estimated decay. Assuming that the GFP molecules in the mother cell are distributed randomly between the daughters, the fluctuations in the numbers of GFP molecules going to each daughter should be binomial distributed, and this has been used previously to infer a conversion factor between GFP molecule numbers and fluorescence levels^24^. In particular, assuming binomial fluctuations, the expectation of the square of the difference $\left\langle {(n_i - m_i)^2} \right\rangle$ should be equal to the total count *n*_*i*_+*m*_*i*_. Given a conversion factor *λ*, such that the GFP molecule counts correspond to (*n*_*i*_, *m*_*i*_) = *λ*(*x*_*i*_, *y*_*i*_), one can estimate *λ* by observing

21 $$1 = \left\langle {\frac{{\left( {n_i - m_i} \right)^2}}{{n_i + m_i}}} \right\rangle = \lambda \left\langle {\frac{{\left( {x_i - y_i} \right)^2}}{{x_i + y_i}}} \right\rangle .$$

**Fig. 9:**
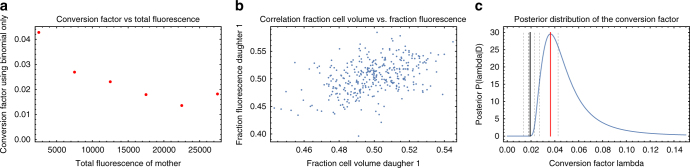
Estimating the conversion factor between fluorescence intensity and number of GFP molecules. **a** Estimated conversion factor *λ*, using the “naive” method which assumes there are only binomial fluctuations, as a function of total fluorescence. Division events were divided into bins based on the mother’s total fluorescence. Bin size was 5000. **b** Correlation between fluctuations in fluorescence and cytoplasm size of two sibling cells immediately after birth. Each dot corresponds to a pair of sister cells with the horizontal axes showing the fraction of the total cytoplasm of the mother and the vertical axis showing the fraction of the total fluorescence of the mother taken up by the first sister. **c** Posterior distribution *P*(*λ*|*D*) of the conversion factor *λ* given the data on our sibling pairs (blue curve). The red line shows the maximum likelihood value λ_*_ = 0.0361. The black lines show the estimated conversion factors that are obtained assuming binomial noise only. The solid line results from using all data, and the dashed lines result from different subsets at different absolute fluorescence values as in the left panel

However, using this “naive” approach, we find that the conversion factor *λ* systematically decreases with total fluorescence (Fig. 9a), changing by as much as fourfold depending on whether division events with low or high absolute fluorescence are used. This result implies that the variance of fluorescence fluctuations grows faster than linear with total fluorescence, suggesting that there are additional fluctuations with variance proportional to total fluorescence squared. Inspection of the data strongly suggests that these additional fluctuations derive from fluctuations in the cell size of the daughters. That is, in addition to the binomial fluctuations there are fluctuations caused by the daughters having unequal size. Practically, cell size at birth is estimated in each daughter cell by extrapolating the linear fit of log(length) as a function of time. Indeed, we observe a substantial correlation between the relative sizes of the siblings and the relative amounts of fluorescence each sibling receives (Fig. 9b, Pearson correlation *r* = 0.44).

We thus developed a more sophisticated model, which takes into account fluctuations in the cell sizes, the binomial fluctuations, as well as measurement noise. For a given division event *i*, let *ρ*_*i*_ denote the measured fraction of the cytoplasm that went to the first daughter, and let *q*_*i*_ = *x*_*i*_/(*x*_*i*_+*y*_*i*_) be the measured fraction of the fluorescence that went to the first daughter. We will assume that *q*_*i*_ is a noisy measurement of the true fraction of molecules *q* = *n*_*i*_/(*n*_*i*_+*m*_*i*_) that went to the first daughter, and that *ρ*_*i*_ is a noisy measurement of the true fraction of the mother’s cytoplasm *ρ* that went to the first daughter. Given *ρ* and a total number of molecules *n* = (*n*_*i*_+*m*_*i*_), the molecule numbers (*n*_*i*_, *m*_*i*_) will show binomial fluctuations and the fraction *q* will have a variance var(*q*) = *ρ*(1−*ρ*)/*n*. In addition to this variance we will assume there is a total measurement noise of variance *v*, so that the total expected square-deviation between the measurements *q*_*i*_ and *ρ*_*i*_ should be *v*+*ρ*(1−*ρ*)/*n*. We will assume that the sum of these fluctuations due to the binomial noise and measurement noise is approximately Gaussian distributed. Finally, we will assume that the binomial variance *ρ*(1−*ρ*)/*n* is well approximated by the measured values *ρ*_*i*_(1−*ρ*_*i*_)/(*λ*(*x*_*i*_+*y*_*i*_)).

Under this model, the probability of observing the fraction *q*_*i*_, given the measured volume fraction *ρ*_*i*_, the conversion factor *λ*, and the total measurement noise *v* is given by

22 $$P\left( {q_i|\rho _i,\lambda ,v} \right) = \left( {v + \frac{{\rho _i(1 - \rho _i)}}{{\lambda \left( {x_i + y_i} \right)}}} \right)^{ - 1/2}{\mathrm{exp}}\left[ { - \frac{{\left( {q_i - \rho _i} \right)^2}}{{2\left( {v + \frac{{\rho _i(1 - \rho _i)}}{{\lambda \left( {x_i + y_i} \right)}}} \right)}}} \right].$$

The log-likelihood of *λ* and *v* is now given by a sum over the *N* division events:

23 $$L(\lambda ,v) = - \frac{1}{2}\mathop {\sum}\limits_{i = 1}^N \frac{{(q_i - \rho _i)^2}}{{\left( {v + \frac{{\rho _i(1 - \rho _i)}}{{\lambda (x_i + y_i)}}} \right)}} + \log \left[ {v + \frac{{\rho _i(1 - \rho _i)}}{{\lambda (x_i + y_i)}}} \right].$$

To obtain the posterior probability of *λ* we marginalize over the unknown variance *v* (using a uniform prior). That is, we calculate $L(\lambda ) = {\mathrm{log}}\left[ {{\int} {\mathrm{exp}}[L(\lambda ,v)]{\rm d}v} \right]$, performing the integral numerically. Using this model, the maximal likelihood value of *λ* is given by

24 $$\lambda _ \ast = 0.0361,$$

and the symmetric 95% posterior probability interval is given by *λ* ∈ [0.026, 0.112].

Figure 9c shows the posterior distribution *P*(*λ*|*D*) obtained with our model. For comparison, Fig. 9c also shows the conversion factors that would be obtained with the naive method that assumes there is only binomial noise, i.e. using all data the number of molecules would be underestimated by almost twofold.

### Data availability

- The designs of the DIMM device, as well as a handbook with detailed experimental methods, are available from Metafluidics web repository at https://metafluidics.org/devices/dual-input-mother-machine/.
- The MoMA software and source code is available on Github: https://github.com/fjug/MoMA. For end users, MoMA is also available as a Fiji plugin at http://sites.imagej.net/MoMA.
- Extensive documentation is provided as a Wiki page containing information about MoMA’s installation and use, as well as tutorial videos: https://github.com/fjug/MoMA/wiki.
- Raw image data of the analyzed growth-channels as well as processed data (estimated cell sizes and fluorescence levels) for all experiments presented in the paper are available from Zenodo at https://doi.org/10.5281/zenodo.746230. A README file is provided with detailed explanation as to which file corresponds to which experiment, and the file format of the processed data files.
- A movie from a time lapse experiment in which *E. coli* ASC622 cells grow in the DIMM under conditions that switch (every 4 h) between glucose and lactose as a carbon source is available on Youtube: https://www.youtube.com/watch?v=2Tznm868fmc. This movie is also available as Supplementary Movie 1.

## Electronic supplementary material

Supplementary Information

Description of Additional Supplementary Files

Supplementary Movie 1
