# Supplementary material for: Monitoring single-cell gene regulation under dynamically controllable conditions with integrated microfluidics and software
Source: Nat Commun. 2018 Jan 15;9:212. doi: 10.1038/s41467-017-02505-0 (PMC5768764; doi:10.1038/s41467-017-02505-0)
Supplement: Supplementary file 1 — Supplementary Information [file 41467_2017_2505_MOESM1_ESM.pdf]

Supplementary Note 1: Data set description and size

For the analysis of the glucose/lactose experiments for which we show data in this article, we accumulated over half a million observations. The precise numbers of growth-channels, observed cell cycles, and total number of individual cell observations for each of our experiments are shown in Supplementary Table 1.

To assemble this dataset, we selected growth-channels that are well filled with cells at the start of the time course, whose cells grow exponentially during the first glucose episode, and which still harbor cells at the end of the experiment. Growth-channels in which most cells get lost were also excluded. We made sure only to include growth-channels that showed no structural defects and furthermore we excluded growth-channels that harbored cells that lysed or showed abnormal elongation. For the experiments reported here, approximately 40% of the growth-channels were discarded because of one or more of these reasons. In addition, we have observed a small reduction in growth rate during the first two hours after the start of illumination, and we therefore discard the first 2 hours of each dataset to allow cells to adapt to the illumination.

Condition	Duration	Growth-channels	# cell observations	# cell cycles
no GFP	24	6	17,283	369
glucose	22	31	92,425	2,061
glucose	22	30	91,470	1,956
lactose	22	30	86,965	1,683
lactose	22	30	86,072	1,704
switch	24	28	76,295	1,455
switch	24	30	88,410	1,632
switch IPTG	16	31	57,166	997

Supplementary Table 1: For each of the 8 experiments we performed, the table lists the condition (described in Table 1 of the main article), the duration of the experiment (in hours), the total number of growth-channels analyzed, the total number of cell observations, and the number of full cell cycles that were recorded.

Supplementary Note 2: Performance of MoMA on datasets from other labs

A problem that is often encountered with image analysis methods that have been developed using data from a pecific system and laboratory is that they fail to generalize well to data produced with other setups in other labs. Because MoMA uses a novel algorithmic approach in which segmentation and tracking are solved as a joint optimization problem, we were confident that the approach should be highly robust and generalize well to data from other settings, but we wanted to check this directly. A challenge is that, currently, virtually no raw time-lapse imaging data from microfluidic devices with mother machine like designs are publically available. However, since the MoMA software had become publically available, a small user-community has been building and we approached several groups from this community to ask whether they would be willing to share some raw time-lapse data produced in their labs, to test MoMA's performance on datasets from other setups.

Several researchers graciously agreed to share some of their data and we obtained datasets from three different labs: A dataset obtained by Benjamin Sellner from the laboratory of Urs Jenal (Biozentrum, Basel), a dataset obtained by Lydia Robert from the Laboratoire Jean Perrin (UPMC university, Paris), and one dataset obtained by Christian Schwall from the laboratory of James Locke (University of Cam-

bridge, UK). Each dataset uses a different microfluidic device, a different strain of bacteria (a different species in on case), and also different growth conditions. Relevant experimental parameters and summary statistics describing each dataset are in Table 2. For reference we provide the same data for our own measurements.

Variable	**Robert**	**Schwall**	**Sellner**	**Kaiser**
Bacterial strain	*E. coli* MG1655	*B. subtilis*	*E. coli* MG1655[pBAD18-mCherry]	*E. coli* ASC662
Growth media	LB	Spizizen's min. media	TB	M9 min. media
Time resolution	4 min.	10 min.	5 min.	3 min.
Num. of growth-channels	3	11	7	216
Total observations	4,798	2,796	4,691	596,472
Full cell cycles	606	322	280	11,859
num. obs. per cell cycle	5.4 ± 1.0	5.2 ± 1.0	10.1 ± 2.7	32.1 ± 11.2
Growth-channel (L $\times$ W; μm)	25×1.2	25×1.6	40×0.9	25×0.8
Flow channel height (μm)	25	50	60	6

Supplementary Table 2: Information about the experimental setup, strains, and growth conditions of the 3 datasets from other labs that we analyzed. Each column corresponds to a dataset, with the name of the person that obtained the data indicated at the top. For reference we also show the corresponding information for our own dataset (Kaiser).

Since experimental conditions were kept constant for all of the 3 datasets, cells were expected to exhibit exponential growth. We processed all data with MoMA and curated the datasets using its interactive graphical interface. Below we characterize the performance of MoMA on these 3 datasets by statistics on the number of curation interactions that were required, as well as by analysis of the quality of the resulting growth curves for individual cells.

Curation statistics

For each growth-channel of each of the 3 datasets (as well as for our own data) we counted the ratio between the total number of curations and the total number of cell observations and denote this as the curation rate of this growth-channel. We then calculated, for each dataset, the reverse-cumulative distribution of curation rate across the growth-channels that were analyzed (Supplementary Fig. 1).

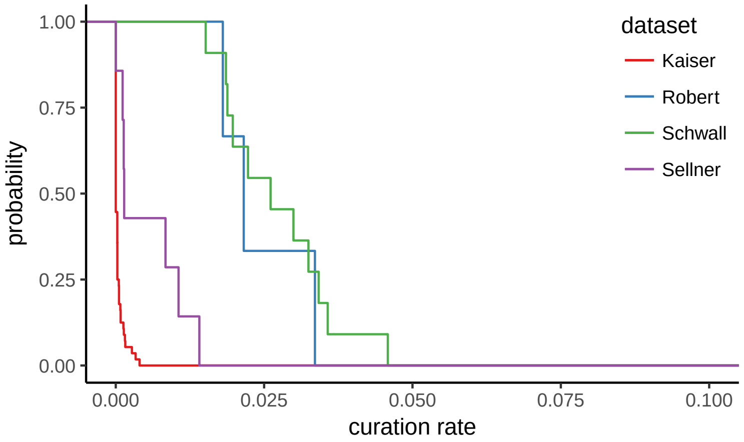

Supplementary Figure 1: Reverse cumulative distributions of the curation rates (number of curations per number of cell observations) for our own data (Kaiser) and for the 3 datasets from other labs.

For our own and the Sellner data there is a large fraction of growth-channels that require essentially no curation and the fraction of frames requiring curation is always below 1%. For the Schwall and Robert datasets most growth-channels do require some interaction, although the fraction is still low, i.e. about 2.5%. Factors that likely affect the curation rate are the number of images per cell cycle (more is better), the extent to which cells can move laterally within growth channels (less is better), and the appearance of the phase contrast images, i.e. whether there is much variability in phase contrast intensity within cells (less is better).

Quality of the growth curves and growth rates of individual cell cycles

After the 3 datasets had been curated, we extracted all full cell cycles and fitted exponential growth curves by fitting the logarithm of cell-size as a function of time. Cell size was taken as height of the bounding-box of the cell as reported by MoMA. We then calculated, for each dataset, the distribution of Pearson squared correlation coefficients of the fits.

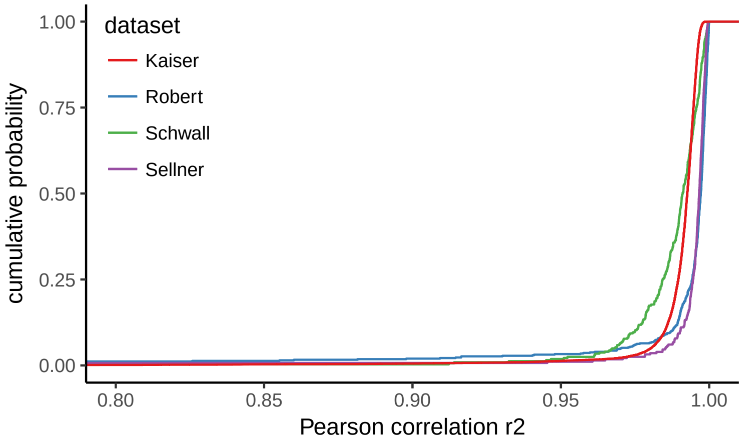

Supplementary Figure 2: Quality of the exponential growth fits for our own and the 3 datasets from other labs. Curves show the cumulative distributions of the Pearson squared correlation coefficients for the linear fits of log-size against time of individual cell cycles. The legend indicates which colored curve corresponds to which dataset.

Supplementary Figure 2 shows that, for all datasets, almost all cell cycles show excellent fits to exponential growth curves, i.e. $r^2 > 0.95$ for all but a very small percentage of the cells, and median r^2 of at least 0.99. This not only shows that cells are growing exponentially in all these datasets, it also shows that MoMA obtains accurate size estimates in all these datasets. Notably, for the large majority of cell cycles, the squared correlation for the Robert and Sellner datasets are even higher than for our own dataset.

We used the fitted exponential growth curves for individual cell cycles to calculate the distribution of growth rates for each of these data-sets (Supplementary Fig. 3).

We see that growth rates vary substantially across the datasets, with the slowest growth rates for our data with cells in M9 minimal media (0.7 doublings per hour on average) and the fastest growth for the cells in LB (2.8 doublings per hour on average). The distribution of growth rates is a simple unimodal distribution for all data-sets. Interestingly, the size of the fluctuations in single-cell growth rates varies substantially across datasets. The coefficient of variation of growth-rates, i.e. standard-deviation divided by mean, is 0.17 for our data, 0.11 for the Robert and Sellner data, and 0.1 for the Schwall data.

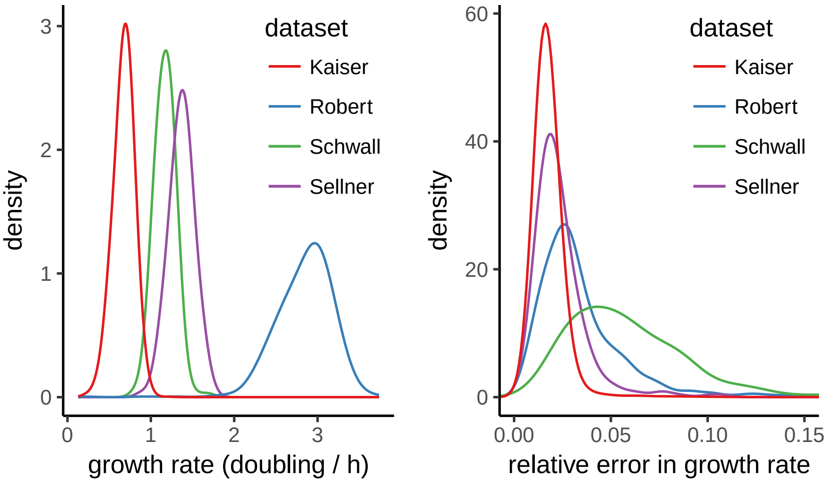

Supplementary Figure 3: Growth rates for individual cell cycles across all datasets. **Left panel:** Distribution of observed growth rates of individual cell cycles for the 3 datasets from other labs and our own data. **Right panel:** Distribution of relative errors on the growth rate estimates for each of the 3 datasets from other labs and our own data.

We also observe that there is some variability in the accuracy of the growth rate estimates across the datasets, with typical relative errors of $1-3\%$ for our data, $1-5\%$ for the data of Sellner, $1-7\%$ for the data of Robert, and $2-10\%$ for the data of Schwall. These differences in accuracy of the growth rate estimates do not stem from differences in the accuracy of the individual size measurements. Indeed, Supplementary Fig. 2 showed that r^2 values of the exponential fits do not substantially differ between datasets. However, as indicated by equation (17) in the main article, the accuracy of the growth rate scales with the inverse of the square-root of the number of observations in the cell cycle. Since there are only around 5 observations per cell cycle for the Robert and Schwall datasets (Supplementary Table 2), the accuracy of the growth-rate estimates is lower for these datasets. This also explains why the Sellner data has slightly higher errors on the growth-rate estimates. In conclusion, if very accurate estimates of the instantaneous growth rates of cells are required, it pays to take phase contrast images frequently.

Supplementary Note 3: Estimating lag times to *lac* operon induction and shut down

In Fig. 2 of the main article, we show results from experiments where the carbon source is alternated between glucose and lactose every 4h with the LacZ-GFP strain. As expected, the expression of LacZ-GFP is induced in each phase of the experiment where lactose is present. To estimate the time until the *lac* operon is induced after a glucose-to-lactose switch in a given cell, we proceeded as follows. First, we checked visually that no cell induces its *lac* operon within the first 9 minutes in all three switches, and used the average of the estimated GFP levels in the first 3 time points after the switch as a measure of the cell's pre-induction GFP level. Second, we also observed that, as soon as the *lac* operon is induced, GFP levels rise quickly and monotonically. We thus defined the induction time of a cell as the first time point where the GFP level had increased significantly beyond fluctuations that could be caused by the cell's auto-fluorescence (which we conservatively took as a fluorescence equivalent of 200 GFP molecules, because the standard-deviation of auto-fluorescence fluctuations in the wild-type MG1655 is equivalent

to the fluorescence of 30 GFP molecules). Thus, in order to successfully observe an induction, we need to observe a cell for at least 4 time points after the switch (3 for estimating the pre-induction GFP level, and at least 1 more to observe induction, i.e. an increase of fluorescence by the equivalent 200 GFP molecules). Of the 1051 cells that were present at the first time point after a switch, 998 were observed for at least 4 time points, and 53 were discarded because they left the channel within the first 4 time points. In order to successfully estimate a lag for as many cells as possible, if a cell divided before inducing, the total GFP of its two daughters was summed and their cell traces considered until the induction threshold was passed, or one of the two daughters was lost (because it left the channel). All in all, the induction lag was successfully quantified for 955 out of 998 cells (the 43 remaining cells left the growth-channel before reaching the induction threshold). We note that the induction time will include the maturation time of the LacZ-GFP fusion proteins.

Estimating the distribution of *lac* induction lags at the first switch

To calculate the probability distribution with error bars for the lag times in the first switch (Fig. 2C of the main article), we proceeded as follows. Our data consisted of $N = 326$ cells that were either observed to induce within 4 hours (312) or that had not induced by the end of the 4 hour lactose phase (14). In addition, there were $M = 24$ cells that were pushed out of the channel before they were observed to induce. We denote by n_k the number of cells that were observed to induce at $3k$ minutes (the cells are imaged every 3 minutes) and, for notational simplicity, we denote the 14 non-inducing cells by $n_{80} = 14$. We will denote by m_k the number of cells exiting the channel (before inducing) at $3k$ minutes. We want to estimate the probabilities ρ_k for a cell to induce at $3k$ minutes with, again for notational simplicity, denoting the probability for a cell not to induce by $240 = 80 \times 3$ minutes by ρ_{80}. Formally, the probability $P(D|\vec{\rho})$ of the observations given a vector of probabilities $\vec{\rho}$ is given by

$$P(D|\vec{\rho}) = \prod_{k=1}^{B} \left[(\rho_k)^{n_k} \left(\sum_{j \geq k} \rho_j \right)^{m_k} \right], \tag{1}$$

with $B = 80$ the number of 3 minute bins. Using a Dirichlet prior $P(\vec{\rho}) \propto \prod_k (\rho_k)^{\lambda-1}$, the posterior estimates and credible intervals for the ρ_k would in principle be obtained by integrals over $P(D|\vec{\rho})P(\vec{\rho})$. However, these integrals are not easily analytically tractable because of the exiting cells. Since the number $M = 24$ of exiting cells is relatively small, i.e. 7% of the data, we decided to use the following approximation. We first determined the maximum likelihood vector of probabilities $\vec{\rho}^*$ by maximizing equation (1). We then replaced our counts n_k and m_k with new 'counts'

$$\tilde{n}_k = (N + M)\rho_k^*. \tag{2}$$

That is, we find the maximum ρ_*^k of the exact likelihood function, and replace our original data with a new dataset in which the M exiting cells are spread over the bins k in proportion with the maximum likelihood probabilities ρ_k^*. The likelihood in terms of these new counts takes on the simple monomial form

$$P(D|\vec{\rho}) = \prod_{k=1}^{B} (\rho_k)^{\tilde{n}_k}. \tag{3}$$

Because the total number of observations is relatively small, we further smoothed the probability distribution $\vec{\rho}$ by averaging counts over 9 minute intervals, i.e. 3 consecutive time points. That is, we define $s_k = \tilde{n}_{k-1} + \tilde{n}_k + \tilde{n}_{k+1}$. Using this, we estimate the fraction f_k of cells with a lag of $3k$ minutes as

$$f_k = \frac{1}{3} \frac{s_k + \lambda}{N + M + B\lambda/3}, \tag{4}$$

where we chose $\lambda = 1/2$ for the parameter of the Dirichlet prior. Using equation (3), the error-bar e_k on f_k is given by

$$e_k = \sqrt{\frac{f_k(1 - 3f_k)}{3(N + M + B\lambda)}}. \tag{5}$$

Similarly to estimating the time to *lac* induction, we can also estimate the time until the GFP signal decreases at the lactose-to-glucose switches by selecting cells that are born before the switch, have been induced during the lactose episode (defined as having at least 400 GFP molecules at the switch back to glucose), and remain in the channel for at least 9 minutes after the switch. Again, if a switching cell divides before the next switch, the total GFP of its two daughters is summed and the cell trace considered until one of the two daughters is lost.

For each cell we measured the delay between the switch and the time at which the GFP level reaches its maximum. We interpret this time to correspond to the sum of the times that it takes to shut down transcription of the *lac* operon, degrade the remaining mRNA, and the maturation time of the last translated LacZ-GFP proteins.

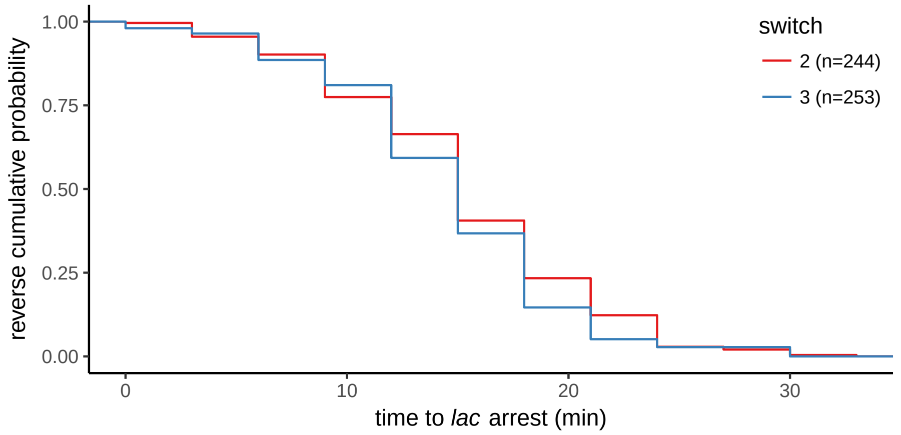

Supplementary Figure 4: Reverse cumulative probability of the delay between the lactose-glucose switch and when the total GFP in the cell starts to decrease. Note that the horizontal axis covers only 35 minutes.

Supplementary Figure 4 shows that the delay times until GFP starts to decrease is short for all cells, i.e. between 10 and 20 minutes, which may well be dominated by the maturation time of the GFP. This strongly suggests that the *lac* operon is quickly shut down in all cells upon the switch to glucose.

Supplementary Note 4: Instantaneous growth rates after *lac* induction

Visual inspection of the growth curves of cells that just induced their *lac* operon and emerge from growth arrest suggest that, instead of a phase where cells grow significantly slower while LacZ-GFP levels are still low, many cells immediately grow at high rates. To quantify this observation we took all cell cycles that fall in the first lactose phase and estimated instantaneous growth rates in a sliding window of 7 consecutive time points (21 minutes of growth) for all points after the lac induction. These instantaneous growth rates for 21 minute windows were then stratified based on the time from the middle of the window

to the time at which the cell (or its ancestor) induced the lac operon. We divided these times into 15 minute bins and calculated the distribution of instantaneous growth rates in each bin as follows.

For each window w we estimate an instantaneous growth rate a_w and an error-bar on this growth-rate σ_w as described in the Methods of the main article. For each bin, we determined the empirical distribution of estimated growth rates a_w (shown as violin plots in Supplementary Fig. 5, left panel) and the standard-deviation of the estimated growth rates (grey lines in Supplementary Fig. 5, left panel). Note that, because each estimated growth-rate a_w has only finite accuracy, part of the observed variation in growth-rates a_w in a bin is due to measurement error. We estimated the 'true' variation in growth-rates in a bin, i.e. correcting for variation due to measurement error, using the following Bayesian procedure. Let α be the true average growth-rate for the windows in the bin, σ the true standard-deviation of the growth-rates, and α_w the true growth-rate of window w. Using a Gaussian prior, the probability that window w will have true growth-rate α_w is given by

$$P(\alpha_w|\alpha,\sigma)d\alpha_w = \frac{1}{\sqrt{2\pi}\sigma} \exp\left(-\frac{(\alpha_w-\alpha)^2}{2\sigma^2}\right) d\alpha_w. \tag{6}$$

The probability to estimate a growth-rate a_w for window w, when the true rate was α_w and the measurement error standard-deviation σ_w is given by

$$P(a_w|\alpha_w,\sigma_w) = \frac{1}{\sqrt{2\pi}\sigma_w} \exp\left(-\frac{(a_w-\alpha_w)^2}{2\sigma_w^2}\right). \tag{7}$$

Integrating over the unknown true growth-rate α_w we then obtain for the probability $P(a_w|\alpha,\sigma,\sigma_w)$ to measure growth-rate a_w given the true average rate α, the true variation σ and the measurement error σ_w:

$$P(a_w|\alpha,\sigma,\sigma_w) = \int d\alpha_w P(a_w|\alpha_w,\sigma_w)P(\alpha_w|\alpha,\sigma) = \frac{1}{\sqrt{2\pi(\sigma^2+\sigma_w^2)}} \exp\left(-\frac{(a_w-\alpha)^2}{2(\sigma^2+\sigma_w^2)}\right). \tag{8}$$

The probability of all the data D for a bin is then given by the product of this expression over all windows w in the bin

$$P(D|\alpha,\sigma) = \prod_w P(a_w|\alpha,\sigma,\sigma_w). \tag{9}$$

Finally, using a uniform prior over both α and σ, we obtain maximum posterior estimates α_* and σ_* by maximizing $P(D|\alpha,\sigma)$ with respect to α and σ. The estimated true means α_* plus and minus σ_* are shown as the black dots and whiskers in the left panel of Supplementary Fig. 5.

The results in the left panel of Supplementary Fig. 5 show that, in the first hour after induction, there is a small but still noticeable reduction in growth rates. Notice however that the shift in growth rates is small relative to their variability. For example, almost a quarter of the cells grow faster than the average growth rate in constant lactose already in the first 15 minutes after their induction. By 60-75 minutes after the switch, the growth rates have assymptoted back to what is observed in constant lactose.

It is reasonable to assume that the small reduction in growth rates in the first hour after induction is due to the fact that LacZ-GFP levels are too low to support growth at full speed. To test this, we again gathered instantaneous growth rates in 21 minute windows, but now stratified by the LacZ-GFP concentration rather than by the time since induction. The LacZ-GFP concentration for a window was estimated as follows. For each time point in the window, LacZ-GFP concentration was defined as the estimated total number of LacZ-GFP molecules divided by the length of the cell, as estimated by MoMA. The average LacZ-GFP concentration in the window was then taken as the average over all time points in the window.

The right panel of Supplementary Fig. 5 shows instantaneous growth rates as a function of LacZ-GFP concentration, calculated in the same way as described above for instantaneous growth-rates as a function

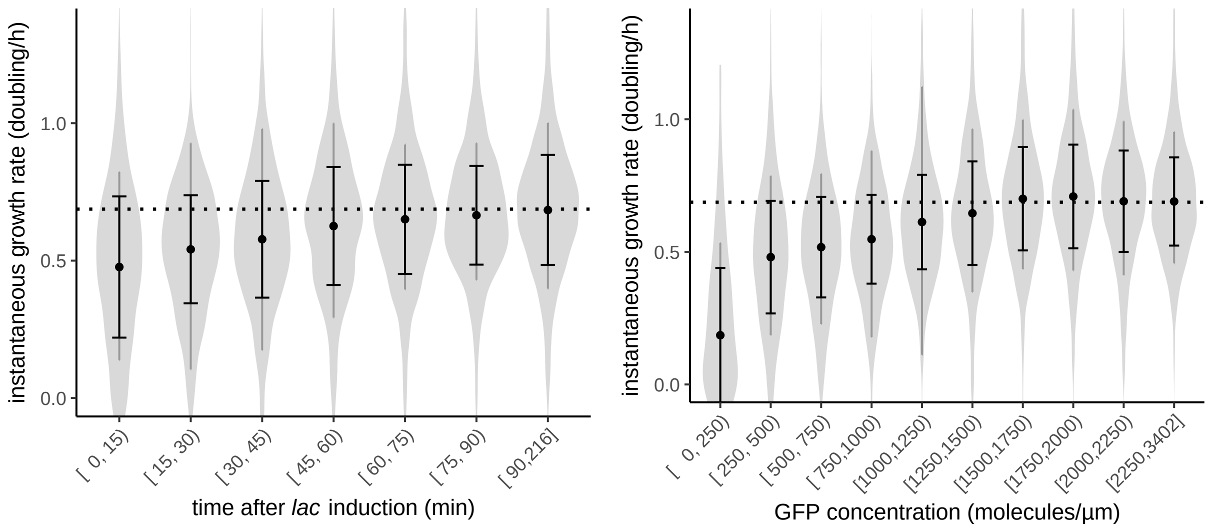

Supplementary Figure 5: Elongation rates as a function of time since induction of the *lac* operon (left panel) and as a function of LacZ-GFP concentration (right panel). Black dots and whiskers show the estimated mean plus and minus standard-deviation of instantaneous elongation rates, i.e. after correction for variance due to measurement error, as a function of time since the *lac* operon was induced (left panel) or as a function of LacZ-GFP concentration (right panel). The grey violin plots and lines show the corresponding raw distributions and standard-deviations of growth rates in each bin. The dotted line corresponds to the average growth rate in constant lactose conditions.

of time after *l*ac induction. For reference, when growing in constant lactose, LacZ-GFP concentration fluctuates approximately between 2000 and 3000 molecules per μm. We see that, below 1000 molecules per μm, there is a small but noticable reduction in growth-rates, and a substantial decrease in growth-rate when levels are more than 10-fold below steady-state levels (i.e. the first bin in Supplementary Fig. 5, right panel). Still, growth-rates vary considerable among cells with similar LacZ-GFP concentration, and many cells exhibit high growth rates even at low LacZ-GFP levels. This shows that cells can sustain high growth rates with LacZ-GFP levels that are up to 10-fold below steady-state levels.

Supplementary Note 5: Determinants of the *lac* induction lag

Memory of *lac* induction at subsequent switches

One unique feature of our setup is that we can trace entire lineages of cells and investigate how the response of a given cell to a change in environment depends on the behavior of all its ancestors. For example, for our particular model system, we can investigate the relationship of the behavior of cells at the second switch from glucose to lactose to the behavior of their ancestor at the first switch.

Supplementary Figure 6 shows a scatter plot with the lag times of cells at the second switch versus the lag time of their ancestor at the first switch 8 hours in the past. We see that *all* cells that show a long lag time at the second switch (i.e. longer than 45 minutes) are descendants of cells that either never induced, or that induced near the end of the first lactose phase. That is, their ancestors never produced substantial amounts of LacZ-GFP. This strongly suggests that, for all cells with ancestors that produced a substantial amount of LacZ-GFP during the first phase in lactose, even after 4 hours of dilution in glucose, the remaining low levels of *lac* operon expression are sufficient to ensure a short lag

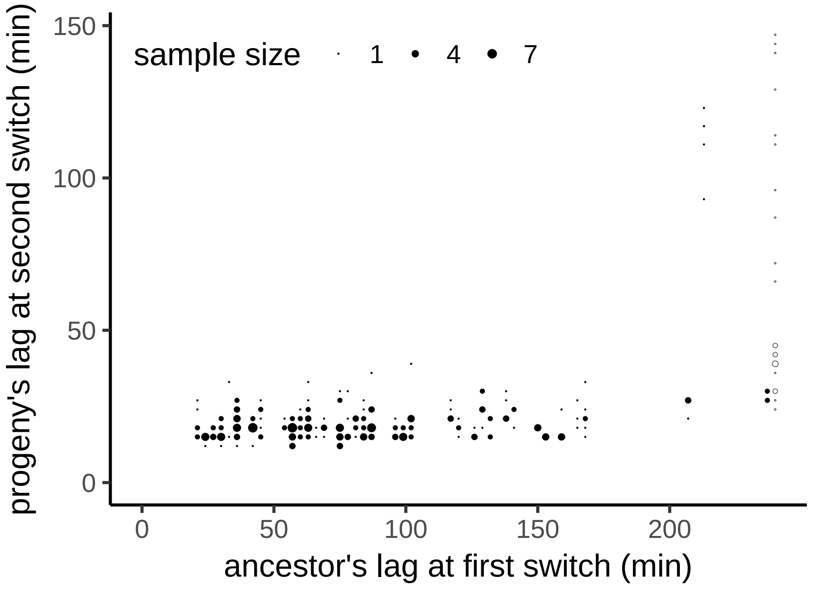

Supplementary Figure 6: Memory of the *lac* induction lag between consecutive switches. Due to the discretisation of measured lag values into 3 minute intervals (imposed by time-lapse acquisition), several pairs have the same values; hence the number of cell pairs observed at a given position is shown by point size. Lags longer than 4 hours (i.e. cells that never exited the lag phase) are shown at the right of the plot with open circles.

at the second switch. That is, lineages of single cells retain a memory of growth in lactose that lasts for at least 4 hours. This memory has previously been observed at the population level [2], but we here show that this is highly reproducible at the single cell level, i.e. *all* descendants of cells that previously grew in lactose retain memory of that growth.

Correlation of *lac* induction lag with physiological state at the switch

We investigated whether the *lac* induction lag depended on the physiological state of the cell at the time of the switch from glucose to lactose. For each cell present at the switch we determined its initial LacZ-GFP level, its growth rate, and its cell cycle phase at the time of the switch. Here we defined the cell cycle phase as the ratio of time since birth to the average total cell cycle duration.

Supplementary Figure 7 shows scatter plots of the observed dependencies between LacZ-GFP level, growth rate, and cell cycle phase and the *lac* induction lag for the first, second, and third switch. The Pearson squared correlation coefficients for the association between each of these variables and lag time are all very small showing that none of these physiological variables is predictive for lag time. The only exception is a small but significant correlation between lag at the second switch and LacZ-GFP level. As explained further in the discussion of Supplementary Fig. 6 below, this correlation results from the fact that the only cells that show long lags in the second switch are descendants of cells that failed to induce in the first switch.

These results are in particular relevant for the first switch, which shows a wide and multi-modal distribution of lag times. The multi-modal distribution of lag times strongly suggests that there is a pre-existing heterogeneity among cells that determines their lag times. However, the results in SupplementaryFig. 7 show that none of the variables we investigated can account for this heterogeneity. It is

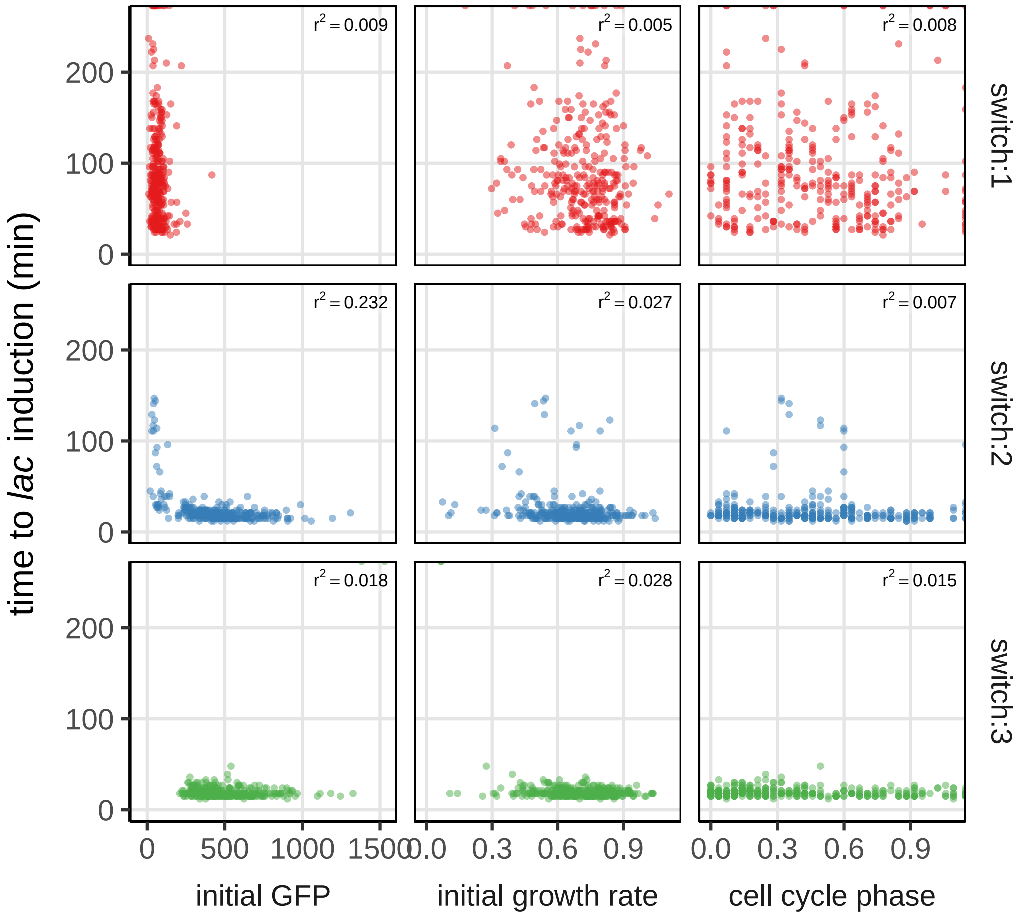

Supplementary Figure 7: Association between *lac* induction lags and the physiological state of the cell at the switch from glucose to lactose. Each panel shows a scatter plot with the association between lag time (vertical axis) and one of three physiological state variables on the horizontal axis. The physiological state variables are LacZ-GFP level (left), growth rate (middle), and cell cycle phase (right). The rows correspond, from top to bottom, to the first, second, and third switch. Pearson squared correlation coefficients are shown at the top right of each panel.

important to note, however, that with the sensitivity of our setup, we can reliably detect cells that have 100 or more LacZ-GFP molecules, but we cannot reliably detect the differences between a cell with zero or, say, 10 molecules of LacZ-GFP.

Heritability and memory of the *lac* induction lag

It is visually striking in timelapse movies (see e.g. Supplementary movie 1 [1]) that the *lac* induction dynamics is more correlated within a growth channel than between growth channels; in other words, once one cell has started to express LacZ-GFP in one growth channel, other cells of the same channel are very likely to also do so before cells of neighbouring channels where induction has not started. This suggests that the non-genetic determinants of the *lac* induction lag are heritable. In order to quantify heritability, we compared the strength of the correlation between the lags in cell pairs with different genealogical relationships: for each pair of related cells undergoing a glucose-lactose switch, pairs are stratified by genealogical relationship and the lag of one cell is plotted against the lag of the other (Supplementary Fig. 8). In this analysis, there were $n = 137$ pairs of sisters, $n = 203$ pairs of cousins, and $n = 265$ pairs of second cousins. How the strength of these correlations varies with the genealogical distance between cells is best seen by computing their correlation coefficient (Supplementary Fig. 8 and inset of Fig. 1G in the main article).

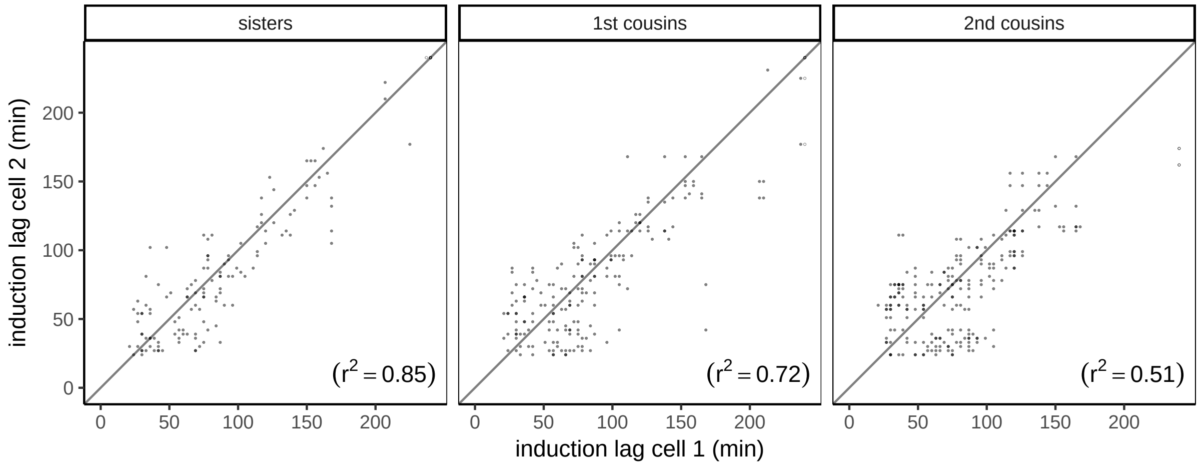

Supplementary Figure 8: Heritability of the *lac* induction lag in naive cells (i.e. at the first switch). Each panel shows a scatter of lags for pairs of sister (left panel), first cousin (middle panel), or second cousin (right panel), with the horizontal axis showing the lag time of the first cell, and the vertical axis showing the lag time of the second cell in the pair. Lags longer than 4 hours (i.e. for cells that never induced the *lac* operon) are shown at the top and right edge of the plot with open circles. Pearson squared correlations are shown in each panel.

We see that lag times show high heritability. Not only is the correlation of lag times in sisters very high ($r^2 = 0.85$), even second cousins, i.e. cells that derive from the same great-grandmother, show highly significant correlation in their lag times. This strongly suggests that lag times are dependent on a heritable epigenetic factor, and that the presence (or absence) of this factor is correlated over multiple generations.

In order to investigate how remarkable the heritability of lag times is, we calculated correlation coefficients between sisters, first cousins, and second cousins, for a number of other physiological traits when growing in constant conditions. These include a number of traits, such as length of the cell at birth,

and LacZ-GFP concentration at birth, that we know must be highly correlated, at least in sisters, because they are directly transmitted from the mother cell. That is, when the mother divides, both daughters are approximately half the size of the mother, and also the LacZ-GFP molecules are approximately equally divided between the two daughters. Indeed, we find that these traits are significantly correlated between sisters ($r^2 \approx 0.5 - 0.65$), and also the growth rates of the individual cell cycles in sisters are clearly correlated ($r^2 \approx 0.5$) (Supplementary Fig. 9). Notably, however, these correlation coefficients are still considerably *lower* than the correlation of lag times between sisters ($r^2 \approx 0.85$). Even more remarkably, none of these physiological traits show clear correlations between cousins or second cousins. That is, correlations in LacZ-GFP concentration and cell size decay within 2 generations. In contrast, lag times show substantial correlations even among second cousins ($r^2 = 0.51$). In summary, lag time to *lac* induction is more strongly correlated among genealogically related cells than any other trait we measured, including ones that we know are directly inherited from the mother, and this correlation lasts for significantly more generations that the correlations of other traits.

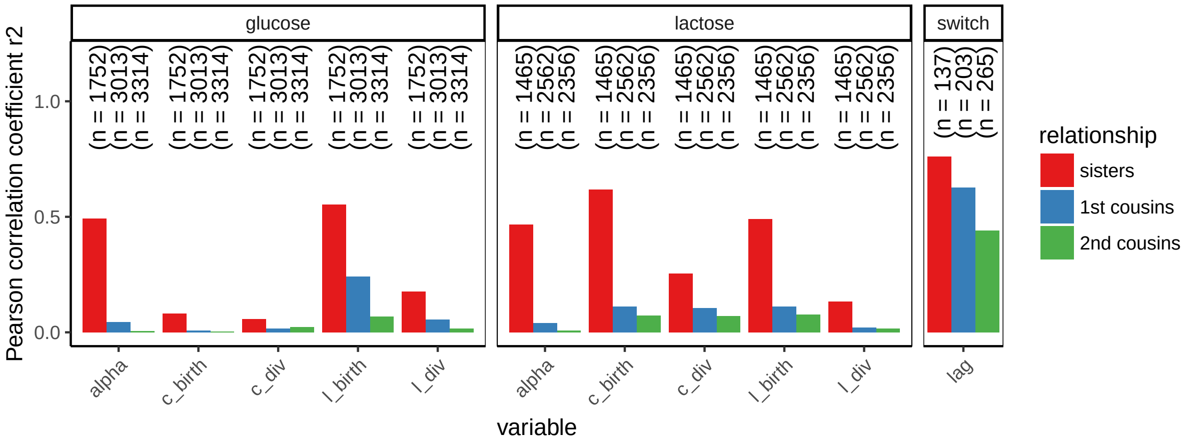

Supplementary Figure 9: Heritability of growth rate (α), cell size at birth (l_{birth}) and at division (l_{div}), and LacZ-GFP concentration at birth (c_{birth}) and at division (c_{div}) for cells grown in constant conditions. In addition, the heritability of *lac* induction lag is shown for comparison (lag). Noticeably, the correlation between sisters is lower at division than at birth, and almost inexistent for LacZ-GFP when its expression is repressed (in glucose).

Supplementary Note 6: *Lac* induction lags with IPTG

We hypothesized that long lag times occur when cells have zero LacY permease and/or zero LacZ-GFP at the time of the switch, because this would prohibit cells either from importing lactose or from metabolizing it into allolactose (which is the molecule that binds the LacI repressor and inhibits its DNA binding). If this were the case, then adding an artificial inducer such as IPTG, which can diffuse into the cell without LacY permease and binds directly to LacI, should abolish the occurrence of long lags.

We tested this by adding 500μM IPTG to the lactose media. Supplementary Figure 10 shows that, with the added artificial inducer, indeed no more long lags are observed. It is interesting to note that the lags with IPTG during the first switch are a bit longer than those seen at the second and third switch with pure lactose. We hypothesize that this is because, at the first switch, most cells have no LacY permease, and thus the IPTG needs to enter the cell by diffusion, which is considerably slower than when LacY permease is available to pump IPTG into the cell. In contrast, at the second and third switch

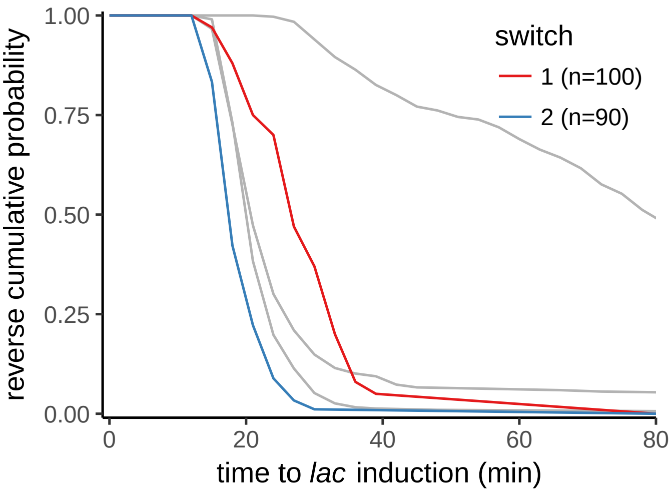

Supplementary Figure 10: Reverse cumulative distribution of lag times until LacZ-GFP induction at the first (red), and second (blue) switch to lactose when 500μM IPTG is added to the lactose containing media. For reference, the lag time distribution at the first, second and third switch with media containing only lactose are shown in grey.

with pure lactose there are still LacY permeases left, and these cause the lactose to be pumped into the cell more quickly. Note also that at the second switch with IPTG, the lag times are even a bit shorter than in the second and third switch with pure lactose. We believe that this speed-up is due to the fact that IPTG is imported at higher rate by LacY than by other non specific transporters. Additionally, the fact that IPTG directly binds LacI, thus circumventing the need of LacZ-GFP metabolizing lactose into allolactose, might also contribute to the shortening of lags.

Supplementary Note 7: LacZ-GFP expression in glucose

Our hypothesis that cells with long lag times correspond to cells that had zero *lac* operon expression at the time of the switch predicts that, while growing in glucose, a substantial proportion of cells (but not all) should have zero *lac* operon expression. To estimate the expression of LacZ-GFP while growing in lactose we obtained the distribution of measured GFP levels for both the strain carrying LacZ-GFP, and the wild-type strain that does not carry GFP (Supplementary Fig. 11).

We see that the median estimated number of GFP molecules in wild-type cells is close to zero, as it should be, given that we have corrected for the average amount of cell auto-fluorescence. In addition, we see that the auto-fluorescence fluctuates from cell to cell with a standard-devation equivalent to about 30 GFP molecules.

The distribution of measured GFP molecules per cells is clearly shifted to slightly higher values in the experiments with the ASC662 strain carrying GFP. We assume that the distribution of the total fluorescence in this strain is a convolution of the distribution of auto-fluorescence of the cells and the distribution of fluorescence originating from GFP molecules. Assuming that these two variables are independent, the mean and variance of the total fluorescence is given by the sums of the means and variances of GFP molecules and auto-fluorescence. We can thus estimate the mean and variance of the distribution of the number of GFP molecules by subtracting, from the mean and variance of total fluorescence, the mean and variance of the auto-fluorescence distribution. Using this approach we estimate

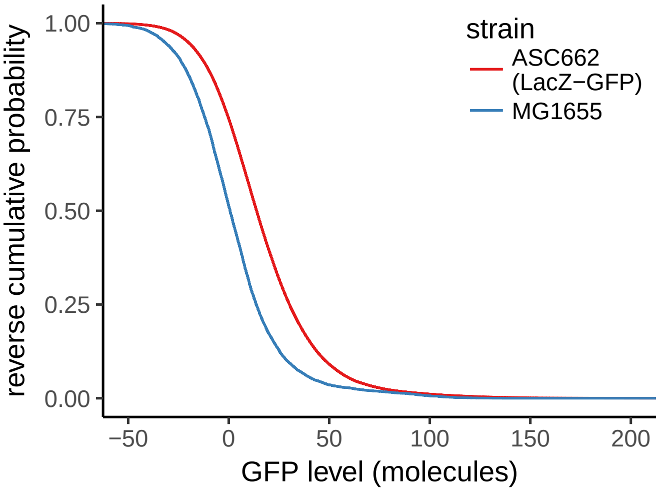

Supplementary Figure 11: Reverse cumulative distributions of the estimated number of GFP molecules per cell while growing in glucose, for the wild-type strain MG1655 that carries no GFP (blue) and the ASC662 strain that carries LacZ-GFP.

that the average number of GFP molecules is $\langle n \rangle = 14$, and that its standard-deviation is $\text{sd}(n) = 10.4$ molecules. Given that the mean is only about one standard-deviation above zero, this would indeed predict that a substantial number of cells have zero LacZ-GFP molecules during growth in glucose.

References

1. *Supplementary movie 1* https://www.youtube.com/watch?v=2Tznm868fmc. Dec. 2015.
2. Lambert, G. & Kussell, E. Memory and Fitness Optimization of Bacteria under Fluctuating Environments. *PLoS Genet* **10,** e1004556 (Sept. 2014).
