# Supplementary material for: Monitoring single-cell gene regulation under dynamically controllable conditions with integrated microfluidics and software
Source: Nat Commun. 2018 Jan 15;9:212. doi: 10.1038/s41467-017-02505-0 (PMC5768764; doi:10.1038/s41467-017-02505-0)
Supplement: Supplementary file 2 — Description of Additional Supplementary Files [file 41467_2017_2505_MOESM2_ESM.pdf]

Description of Additional Supplementary Files

File Name: Supplementary Movie 1
Description: Time lapse of E. coli ASC662 cells (MG1655 LacZ-GFP) growing in the DIMM device under conditions that switch every 4 hours between M9+0.2% glucose and M9+0.2% lactose. The current condition is shown in the coloured bar at the top, the time is indicated by the clock at the top right and the vertical length scale indicates 5µm. Phase contrast and GFP channels are acquired every 3 minunutes and shown as a composite image (after mild saturation for better rendering).
